# The Pattern of Genetic Variability in Apomictic Clones of *Taraxacum officinale* Indicates the Alternation of Asexual and Sexual Histories of Apomicts

**DOI:** 10.1371/journal.pone.0041868

**Published:** 2012-08-01

**Authors:** Ľuboš Majeský, Radim J. Vašut, Miloslav Kitner, Bohumil Trávníček

**Affiliations:** Department of Botany, Faculty of Science, Palacký University, Olomouc, Czech Republic; University of Massachusetts, United States of America

## Abstract

Dandelions (genus *Taraxacum*) comprise a group of sexual diploids and apomictic polyploids with a complicated reticular evolution. Apomixis (clonal reproduction through seeds) in this genus is considered to be obligate, and therefore represent a good model for studying the role of asexual reproduction in microevolutionary processes of apomictic genera. In our study, a total of 187 apomictic individuals composing a set of nine microspecies (sampled across wide geographic area in Europe) were genotyped for six microsatellite loci and for 162 amplified fragment length polymorphism (AFLP) markers. Our results indicated that significant genetic similarity existed within accessions with low numbers of genotypes. Genotypic variability was high among accessions but low within accessions. Clustering methods discriminated individuals into nine groups corresponding to their phenotypes. Furthermore, two groups of apomictic genotypes were observed, which suggests that they had different asexual histories. A matrix compatibility test suggests that most of the variability within accession groups was mutational in origin. However, the presence of recombination was also detected. The accumulation of mutations in asexual clones leads to the establishment of a network of clone mates. However, this study suggests that the clones primarily originated from the hybridisation between sexual and apomicts.

## Introduction

Asexual reproduction through seeds (i.e., apomixis) occurs in less than 1% of flowering plants [1]. Although asexual organisms are expected to be evolutionary dead ends [2], apomictic plants are known to occur in numerous phylogenetic groups across all flowering plants [3]. In some genera, such as the genus dandelion (*Taraxacum*), the widespread distribution of apomictic clones suggests that they are temporarily ecologically successful [4]. Asexual reproduction thus offers a low-cost alternative to sexual reproduction. Although apomixis gives plants temporal ecological and evolutionary benefits, sexuality is generally playing the dominant role in their reproduction, a concept that is referred to as the *Paradox of Sex* [5].

The lack of recombination is expected to direct apomicts towards their extinction [2]. The most ancient asexuals are found among bdelloid rotifers and darwinulid ostracods, which appear to have persisted for tens of millions years [6]. However, the age of genes involved in the regulation of apomixis and their evolutionary origins are still unknown. The majority of plant apomicts are polyploids of putative young Pleistocene origin, with diploid sexuals as their closest relatives [3], [7], [8]. The association of polyploidy and apomixis may be a consequence of asynchronous expression of duplicate-genes controlling megagametogenesis, which causes regular meiosis to malfunction [8] and/or process of hybridisation and polyploidisation might favour mutation, leading to parthenogenesis to avoid sterility or loss of fitness in hybrids [1]. The spread of apomicts could occur directly through apomictically raised seeds or indirectly through pollen, when genes for apomixis are transferred into new genetic backgrounds derived from sexuals. Gene flow among apomicts and sexuals keeps apomictic genes present for long periods of time, allowing the genes to avoid mutation and thus decreasing the mutation load [1], [4], [9].

Apomicts have significant advantages over sexuals in colonising new areas [2]. A vast majority of asexuals occur over wide areas, e.g., *Taraxacum, Hieracium, Rubus,* and *Poa*, with significant geographic parthenogenesis in their distribution [4], [10]. Another advantage of apomixis is the high proportion of loci fixed in heterozygous conditions compared to that of sexuals [11], [12]. In contrast, asexuality has far-reaching penalties, such as a lack of diversity, the limited possibility of acquiring heritable variability (e.g. [13]) and an increased mutation load leading to the extinction of clones [4], which give apomicts an adaptive disadvantage. However, the short-term advantages of apomixis have become of interest to the agricultural industry. Fixing the heterozygous genetic condition of plants via apomixis and revealing the nature of the genetic control of apomixis are important goals for plant breeding research [14].

The genus *Taraxacum* Wigg. (*Asteraceae, Cichorioideae*) consists of perennial herbs that are widely distributed throughout the world (with exception of Antarctica). The putative centre of origin is in Central Asia in a region that includes the Himalayas [7]. Apomictic dandelions in Europe are believed to be of young evolutionary origin [15], with an explosive spread in the late Holocene period [7]. Apomixis in *Taraxacum* is obligate meiotic diplospory, which is the type that is most similar to sexual reproduction among apomixis systems [3]. Diplosporous plants undergo part of meiosis, in case of meiotic diplospory the anaphase II during megasporogenesis (spore formation from the Megaspore Mother Cell) is skipped resulting in development of two unreduced megaspores (unlike to sexual reproduction that results in development of four reduced megaspores). In both, sexual and diplosporous plants one of the megaspores further mitotically divides to form an embryo sac (megagametophyte). Aposporous plants differ from both above types in formation the embryo sac directly from cells of sporophyte by mitosis (i.e., meiosis is completely omitted). In dandelions, apomictic reproduction is regulated by three dominant loci [16], (Vašut, unpublished results). Two loci are already identified–*DIPLOSPOROUS* (*DIP*) and *PARTHENOGENESIS* (*PAR*) [9], [17], [18].

Species of *Taraxacum* form polyploid series; apomicts are mostly triploids or tetraploids, whereas sexuals are mostly confined to diploids [19], [20]. Sexual species show extensive reticulate evolution, which was detected in apomicts as well [15], [21], [22]. *Taraxacum* species are classified into morphological groups (sections) that contain one or more sexual species and polyploid clumps of apomictic accessions (traditionally either classified as microspecies or not recognised). Apomicts are either of autopolyploid origin or are the result of hybridisation [19]. Therefore, apomicts have the potential to reveal evolutionary processes within the genus in detail.

Fully asexual or mixed sexual apomictic *Taraxacum* populations comprise individuals with extended genotypic variability; such genotypes of these populations can be widely distributed or can exist as local clones [23]–[27]. Sexual recombination and mutational differentiation are considered to be main sources of this variability [24], [25]. Interploidy gene flow can occur within populations [28], [29]. This gene flow was suggested to be responsible for the presence of shared allozyme polymorphism and unique alleles present in populations of different cytotypes [23] and for the spatial structure of cytotype distribution [27]. Only a few studies have examined the variability within clones or within morphologically uniform accessions (e.g. [30], [31]). Mes *et al.* [32] used Internal Transcribed Spacer (ITS), amplified fragment length polymorphism (AFLP) and simple sequence repeat (SSR, microsatellites) markers to characterise apomictic clones from sect. *Naevosa.* He stressed that individuals from a single clone with sufficiently long asexual histories may differ genetically due to mutation accumulation.

We consider that the apomictic clones are genotypes that underwent only clonal reproduction since their most recent common ancestor, and thus, genotype diversity within the clone has a detectable mutational background. There are two different approaches to the analysis of population genetics of apomictic plants (e.g. [25], [32]): i) plants are sampled randomly from the population, and ii) a plant sample is selected based on morphological criteria (e.g. [33], [34]). In this study, we adopted the second approach and applied it to nine apomictic accessions of *Taraxacum officinale* agg. sampled in regions of sympatric occurrence of sexuals and apomicts, allowing us to study the mutation load and the formation of novel genotypes in greater detail. Morphology was the key criterion for accession assignment. In present study, we addressed following questions: 1) What is the pattern of genotypic variability within and differentiation among apomictic clones? 2) What is the source of intraclonal and interclonal variability? 3) What is the origin of apomictic clones? 4) What is the detection ability of molecular markers? To answer these questions, we used three types of molecular markers. Microsatellites are flexible tools for population studies (e.g. [12], [35]–[37]). The development of microsatellites for *Taraxacum* [38], [39] enabled them to be used in population studies (combined with other markers) to detect clones and to investigate population structures and gene flow (e.g. [25], [32]). Microsatellites are suitable for population genetics due to their polymorphism, high mutational rate and co-dominant nature (e.g. [40], [41]). In contrast, dominant AFLP markers are firmly established as valuable tools for a wide range of evolutionary and biosystematic studies (e.g. [42]–[45]). Both types of marker systems are commonly used for measuring population genetic structure and diversity, providing congruent and robust results [46]–[50]. To compare the fingerprints acquired from nuclear markers with information that is inherited matrilineally, the *trnL-trnF* region in cpDNA was sequenced.

## Materials and Methods

### Plant Material and DNA Extraction

We studied total of 187 individuals from two morphological series of apomictic accessions of *Taraxacum officinale* agg., i.e., *T*. sect. *Taraxacum* (syn. *T*. sect. *Ruderalia*) [51], [52]. The first group comprises a complex of six morphologically closely related accessions (*T. amplum* agg. – *AMP* group, each phenotyped accession denoted as *amp1*– *amp6*); the second one contains three morphologically divergent accessions (*OSP* group, denoted as *O*, *S*, *P*). Term “accession” is used in sense of morphologically homogeneous phenotypic unit. All accessions were phenotyped according to the taxonomic microspecies concept and only confirmed phenotypes were included in this study; a complete list of the individuals studied (including taxonomic identification) is provided in Table S1.

**Figure 1:**
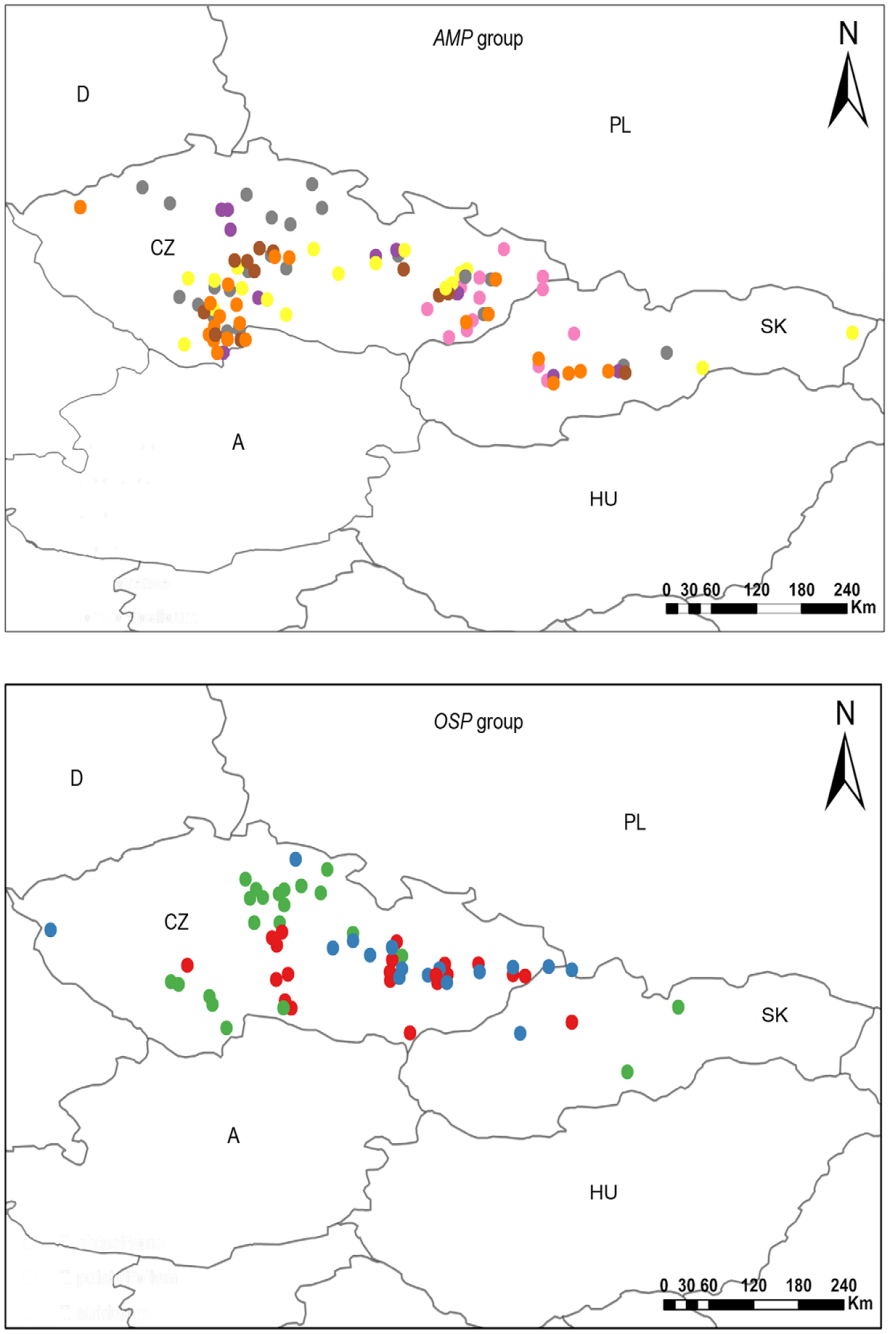
Geographical distribution of studied individuals of apomictic *Taraxacum* accessions. *AMP*-group: yellow circle – *amp1*, orange – *amp2,* purple – *amp3,* brown – *amp4,* pink – *amp5,* grey – *amp6*; *OSP*-group: red circle – *O*, green – *S*, blue – *P*. Country codes: A – Austria; CZ – Czech Republic; D – Germany; HU – Hungaria; PL – Poland; SK – Slovakia. For taxon abbreviations see Table S1.

We sampled one individual per one locality across the wide geographic range of Central Europe (Figure 1). The plant material was documented by depositing herbarium specimens into herbarium of the Department of Botany, Palacký University in Olomouc, Czech Republic (*OL*).

Apomictic reproduction was confirmed by either emasculation or Flow Cytometric Seed Screen (FCSS) [53]. To sequence the *trnL-trnF* region, four sexual diploid plants were added. Ploidy levels were confirmed by flow-cytometric analysis of relative DNA content using an inner diploid control.

Genomic DNA was extracted from voucher specimens or fresh leaves, following CTAB (Cetyl Trimethyl Ammonium Bromide) protocol of Doyle & Doyle [54] with minor modifications.

The research did not involve any endangered or protected plant species, originating from restricted privately-owned or protected territories, so no specific permits were required for plant sampling.

### Microsatellite Genotyping

All 187 individuals were genotyped for six microsatellite loci: MSTA44B, MSTA53, MSTA78 [38]; and MSTA93, MSTA131, MSTA133 [39]. The PCR amplifications were performed in total volume of 15 μl with 0.2 μM of each primers, 0.2 mM dNTPs, 1X PCR reaction buffer (containing 1.5 mM of MgCl_2_ in final volume) and 0.42 U of GoTaq DNA Polymerase (Promega). Detailed information on PCR conditions (e.g. annealing temperature, number of cycles) is available upon request.

### AFLP Fingerprinting

Ninety-six individuals including all nine apomictic accessions (see Table S1) were analysed by AFLP (Amplified Fragment Length Polymorphism) for three primer combinations (*Eco*RI-AGC/*Mse*I-CAAT, *Eco*RI-AAT/*Mse*I-CAAC, and *Eco*RI-AGC/*Mse*I-CGATG). AFLP analyses followed the protocol of Vos *et al.* [55] with the modifications of Kitner *et al.* [56].

### cpDNA Sequencing and Sequence Alignment

The *trnL-trnF* region was sequenced in four samples from each apomictic accession and in the diploid sexual. A PCR reaction was performed in a total volume of 25 μl with 10 ng of template DNA, 2 μM of *e* and *f* primers [57], 0.2 mM dNTPs, 1X PCR reaction buffer (containing 2 mM of MgCl_2_) and 1 U of *Pfu* DNA Polymerase (Fermentas). The reaction conditions were as follows: 95°C for 2 min; 30 cycles with 95°C for 1 min, 52°C for 1 min, and 72°C for 1 min; followed by 5 min at 72°C. The PCR products were sequenced using an Applied Biosystems 3730xL capillary sequencing system. Sequences were edited in BioEdit [58], alignment and haplotype identification was performed in Mega 5 [59]. Sequence data are deposited in GenBank (accession numbers JQ696774-JQ696810).

### Fragment Analyses of Microsatellite Genotypes and AFLP Profiles

PCR products were separated by denaturising polyacrylamide gel electrophoresis (PAGE) and visualised by silver staining. The 30–330-bp AFLP® DNA ladder (Invitrogen) was used to size the microsatellite alleles. If only one allele was observed for a locus, then the individual genotype was considered to be an absolute homozygote; when two different alleles were observed, the third was coded as missing data. In the case of AFLP, the profiles were visually checked and coded as a binary matrix. To avoid the genotyping errors and to retain reproducibility of the analyses, several control levels were included during the entire study: blind samples, double samples and repetitions. This allowed estimation of the error rate, which was calculated as the difference between all markers and the markers used in the final matrix.

### Microsatellite Data Analyses

Microsatellites data were analysed as microsatellite genotypes based on the number of repeats. They were also scored using a binary matrix, where the presence/absence of a fragment of a particular size was coded as 1/0. This approach was used to perform hierarchical AMOVA with Arlequine 3.5 [60] based on pairwise genetic distances. To determine whether differences among genotypes are due to mutations or recombination, a character compatibility test was performed using module Jactax from package Pica 4.0 [61]. Character compatibility test is based on the assumption that pairs of loci should have fully compatible variation in the absence of recombination. If the ancestral condition for a pair of binary loci is 00, under the assumption of clonality only two of the three possible character states might be expected: 01 and 11. Presence of the fourth character state (10) is evidence of incompatibility and suggests the action of recombination. Incompatibility is inferred when all four combinations of two binary states are observed within matrix. In compatibility analysis the number of incompatibilities (*MIC* – the matrix incompatibility count) between each pair of multilocus genotypes is computed. After removing of genotypes with the highest number of incompatibilities only fully compatible genotypes should be left in the matrix differing only due to mutations (*MIC*  = 0) [62], [25].

From genotype data several locus and multilocus statistics were computed using SPAGeDi software [63]: the number of genotypes (*NG*), overall number of alleles, the number of different alleles (*NDA*), the means allele size (*MAS*), the range of allele size, and gene diversity (*G_e_/G_e_*). To characterise polymorphism and among-population differentiation, locus and multilocus estimates of *F*- and *R*-statistics were calculated. The contribution of stepwise mutations (SMM) vs. nonstepwise mutations (IAM – infinite allele model) to population differentiation was tested (i.e., whether the observed *R*_ST_ is significantly larger than its value after permuting allele sizes among alleles within populations) [41]. P values were obtained after 999 random permutations.

### AFLP Data Analyses

Basic population statistic indices such as the mean number of bands (*NB*) and the number of polymorphic bands (*NPB*) at the 5% level, number of private markers (*PrB*; restricted to a given population), number of diagnostic markers (*DB*; present in all individuals in a population) were calculated in Famd [64]. Polymorphism (*P*), Nei’s gene diversity (*H_j_*), number of different genotypes (*NG*) and genotype diversity (*GD*) were calculated using the R-script of AFLPdat [65].

To evaluate the distribution of genotypic variation, AMOVA was performed as described for SSRs. To explore the relationship within and among groups, Principal Coordinate Analysis (PCoA) was performed in NTSYS-pc version 2.02 [66] (Jaccard similarity matrix), and inspected on a 3D plot. An unrooted neighbour-joining tree (Dice coefficient of similarity) was constructed by FreeTree [67] and visualised in TreeView [68] (bootstrap support with 1,000 replications; [69]). Computing of split network based on SplitDecomposition method (uncorrected *P*-distances, Hamming distances), was done in SplitsTree 4 ([70], robustness tested by 1,000 bootstrap replicates).

To determine different genetic groups, Bayesian clustering approach was used as implemented in the programs Structure 2.2 [71] and Baps 3.2 [72]. The difference between Structure and Baps is in the treatment of *K* (number of clusters). Whereas Structure uses the Markov Chain Monte Carlo (MCMC) algorithm to cluster genetically similar individuals and to estimate the likelihood of the data for different numbers of groups (*K*), in Baps frequency of alleles and the number of genetically different groups are taken as random variables and the program estimates one optimal partitioning. Computation in Structure was set up for the recessive allele model and the admixture model with correlated allele frequencies. The *K* was set to 1–11 with 10 replicate runs for each *K* using the 1,000,000 MCMC iterations following the period of 100,000 burn-in iterations. The computation was carried out on the freely accessible Bioportal of the University of Oslo (www.bioportal.uio.no). The R-script Structure-sum-2009 [73] was used to summarise the output files: calculation of similarity coefficients between replicate runs (*SC*), means of the posterior log probability [mean*L*(*K*)], and a quantity based on the second order rate of change of the likelihood function with respect to *K* (Δ*K*) (as denoted in [74]). Additionally, two programs, Clumpp [75] and Distruct [76], were used to summarise the Structure outputs and to figure the clustering graphically. For analyses in Baps, module “clustering of individuals” was used. *K* ranged from 1 to 11, and the analysis was repeated ten times.

## Results

### Microsatellites

The total number of scored alleles over six microsatellite loci was 2842 in 186 individuals. The ranges of allele sizes and allele numbers per locus are summarised in Table 1. Different levels of control (including repeated PCRs and double samples) confirm high reproducibility of microsatellites data (error rate <1%). The number of different alleles and genotypes observed per locus was low; the range was between 8 (MSTA133) and 16 alleles (MSTA44B). Low numbers of SSR genotypes were detected with high levels of allele sharing across investigated accessions. The majority of different alleles observed per loci in all six *AMP* accessions were also present within genotypes of *amp1*. However, the genotypes were always accession specific (except for locus MSTA131, *amp2* and *amp4* shared the same genotype). Most of genotypes were clonal or differ in only one or two alleles in a few repetitions. No variability was observed among all genotyped individuals of *S*, with only one genotype detected over all of the loci. For *O*, *P*, *amp4*, two genotypes were observed for only one locus, and all other loci were without genotype variation. The rest of the species show higher genotype variability within genotyped loci. Individuals of *amp1* showed the highest genotype and allelic variability (Table 2). The overall gene diversities *G_e_* and *G_e_* within the dataset were high and very similar over all loci, from *G_e_*  = 0.81 (loci MSTA133, MSTA53) to *G_e_*  = 0.89 (MSTA44B) (Table 1, Table 2). However, in loci MSTA93 among *P* and for MSTA133 within *amp4*, only one allele was detected; thus, *G_e_*  = 0 (Table 2).

**Table 1:** Allelic diversity of six nuclear microsatellite loci for *Taraxacum*.

All populations						
Locus	Allele size	*K*	*G_e_*	*F_is_*	*F_st_*	*R_st_*
MSTA131^1^	167–203	10	0.8185	−0.5908***	0.2655***	0.2983***
MSTA133^1^	260–312	8	0.8051	−0.5397***	0.2924***	0.4879***
MSTA53^2^	228–234	11	0.8122	−0.5402***	0.3070***	0.4230***
MSTA44B^2^	165–199	16	0.8933	−0.5690***	0.3065***	0.2169***
MSTA78^2^	150–182	12	0.8383	−0.5397***	0.2534***	0.4415***
MSTA93^1^	278–317	10	0.8151	−0.4108***	0.5501***	0.3385***

Allele size, size range of PCR products in number of nucleotides; *K*, total number of alleles; *G_e_*, gene diversity; *F*_IS_, Wright’s inbreeding coefficient; *F*_ST_, relative differentiation based on allele identity; *R*_ST_ relative differentiation based on allele size.

*** – significant value, P<0.001.

1 [39].

2 [38].

**Table 2:** Descriptive statistics for nine apomictic *Taraxacum* accessions based on six SSR loci. For taxon abbreviations see Table S1.

	*amp1*						*amp4*						*O*					
*N*	23						12						21					
	MSTA133	MSTA131	MSTA93	MSTA78	MSTA53	MSTA44B	MSTA133	MSTA131	MSTA93	MSTA78	MSTA53	MSTA44B	MSTA133	MSTA131	MSTA93	MSTA78	MSTA53	MSTA44B
***MAS***	32	65,1	121,2	66	41,2	68,9	31	64	127,5	65,5	42,3	67	30,5	68,3	124	63,4	44,3	66,5
***NDA***	6	7	6	8	8	10	1	2	2	2	4	3	2	3	2	4	3	2
***NG***	9	7	7	7	7	8	1	1	1	1	2	1	1	1	1	2	1	1
**G*G_e_***	0,7582	0,7262	0,677	0,648	0,603	0,7362	0	0,5217	0,522	0,522	0,703	0,6857	0,5122	0,6774	0,512	0,693	0,677	0,5122
	***amp2***						***amp5***						***S***					
***N***	**23**						**14**						**23**					
	**MSTA133**	**MSTA131**	**MSTA93**	**MSTA78**	**MSTA53**	**MSTA44B**	**MSTA133**	**MSTA131**	**MSTA93**	**MSTA78**	**MSTA53**	**MSTA44B**	**MSTA133**	**MSTA131**	**MSTA93**	**MSTA78**	**MSTA53**	**MSTA44B**
***MAS***	30	64	120,6	67,8	41,1	65,3	31	69,6	123,4	67	43,6	68,7	28	68,7	120,3	63	42,3	72,5
***NDA***	5	2	4	4	3	5	4	4	4	3	4	3	3	3	2	3	3	2
***NG***	2	1	3	3	2	2	3	2	3	2	2	2	1	1	1	1	1	1
***G_e_***	0,6952	0,5111	0,553	0,56	0,532	0,6952	0,5751	0,5645	0,575	0,553	0,698	0,5416	0,6765	0,6765	0,451	0,677	0,677	0,5111
	***amp3***						***amp6***						***P***					
***N***	**18**						**36**						**17**					
	**MSTA133**	**MSTA131**	**MSTA93**	**MSTA78**	**MSTA53**	**MSTA44B**	**MSTA133**	**MSTA131**	**MSTA93**	**MSTA78**	**MSTA53**	**MSTA44B**	**MSTA133**	**MSTA131**	**MSTA93**	**MSTA78**	**MSTA53**	**MSTA44B**
***MAS***	31,1	66,5	120,6	65,3	41,9	67	29,5	70,1	124,8	67	41,5	67,7	28,7	68,3	124	62,7	45	62,5
***NDA***	5	3	3	3	4	5	2	6	4	6	3	6	3	3	1	3	3	2
***NG***	3	2	2	1	3	2	2	4	4	5	2	3	1	1	1	1	2	1
***G_e_***	0,7303	0,679	0,532	0,679	0,584	0,7146	0,5066	0,5647	0,183	0,702	0,44	0,7024	0,68	0,68	0	0,68	0,544	0,5152

*N*, sample size; *MAS*, mean allele size in number of repetition of repeat motif; *NDA*, number of different alelles; *NG*, number of different genotypes; *G_e_*, gene diversity counted for loci.

The values of *R*_ST_ and *F*_ST_ were similar (Table 1, Table 3). However, the test of significance of mutational model favours IAM model and the use of *F*_ST_ for description of variability for multilocus and majority of locus estimates (data not shown). Nevertheless, locus estimates for MSTA133 (P<0.05) and MSTA78 (P  = 0.059) within *AMP+OSP* and MSTA78 (P<0.01) within *OSP* suggested that some loci may undergo also stepwise mutations. As expected from the nature of the plants used in this study, the inbreeding coefficient *F*_IS_/*R*_IS_ reached high negative values. In multilocus estimates, *F*_IS_/*R*_IS_  =  −0.5400/−0.5744 for the *AMP+OSP* group (Table 3). A similar situation was observed for locus estimates (Table 1). The values of *R*_ST_ and *F*_ST_ were similar, and high (P<0.05; Table 1, Table 3), suggesting that the majority of SSR diversity is present among apomictic accessions. The multilocus values for the *AMP+OSP* dataset were *F*_ST_/*R*_ST_  = 0.3293/0.3076 (Table 3).

**Table 3:** Multilocus estimates of *F-* and *R-*statistic for all six microsatellite loci for studied apomictic *Taraxacum* accessions.

	F-statistics		R-statistics	
	*F_is_*	*F_st_*	*R_is_*	*R_st_*
All Loci [*AMP*+*OSP*]	−0.5400***	0.3293***	−0.5744***	0.3076***
All Loci [*AMP*]	−0.5147***	0.2881***	−0.5616***	0.2132***
All Loci [*OSP*]	−0.6126***	0.3534***	−0.6706***	0.5935***

*** – significant value, P<0.001.

No monomorphic alleles were observed for loci MSTA78 and MSTA53 across the analysed samples, which should be linked the *DIP* locus. AMOVA showed that 88.4% of the variation was present among apomictic accessions and only 11.6% within accessions. When one hierarchical level is added (*AMP*, *OSP*), the pattern of the variation remains unchanged with 22.9% of variation between *AMP/OSP* groups.

A character compatibility test was applied on only *amp1*, *amp2*, *amp3*, *amp5*, *amp6*, *S*, *OSP*, and *AMP*. For others apomictic accessions, fewer than four required genotypes were present (Table 4). In *amp5* and *S*, no incompatibility (no genotypes, that would be in disagreement with fully asexual differentiation) was found, and in *amp2*, *amp3*, *amp6*, only one genotype cause matrix incompatibility; after its removal, *MIC*  = 0 (Table 4). In the *OSP* group, two genotypes had to be removed to *MIC*  = 0. For *amp1*, 9 genotypes caused 201 incompatibilities. Investigation of the whole *AMP* group led to the deletion of 42 genotypes from a total of 46 to reach *MIC*  = 0 (Table 4). This result appears to be inconsistent with only mutational differentiation and a fully asexual history of the genotypes.

**Table 4:** Character compatibility test for studied apomictic *Taraxacum* accessions.

	*amp1*	*amp2*	*amp3*	*amp4*	*amp5*	*amp6*	*O*	*P*	*S*	*AMP*	*OSP*
*N*	23	23	18	12	14	36	21	17	23	126	61
*NG*	15	5	8	2	8	8	2	3	4	46	9
*MIC*	201	5	2	-	0	16	-	-	0	743	51
*E*	6	4	7	-	8	7	-	-	4	4	7

*N*, number of samples; *NG*, number of genotypes; *MIC*, matrix incompatibility count; *E*, number of genotypes left at *MIC*  = 0. For taxon abbreviations see Table S1.

### AFLPs

Three primer combinations produced a total of 162 unambiguously scorable markers, of which 129 were polymorphic. The error rate corresponds to 2%. An observation of clonality (allowed difference of three markers, calculated from error rate: 2%  = 3.24) showed that most of the clonal genotypes in *O* and *S* had one AFLP phenotype among ten individuals, while the most diverse in *amp3* and *amp1* had 10 different AFLP phenotypes observed among eleven individuals (Table 5). The highest *GD* was detected (with both markers) in the *amp1* accession, and among ten AFLP phenotypes, only seven also had different SSR genotypes. In *amp3*, ten AFLP phenotypes were detected, but only four also had different SSR genotypes. The difference occurred in one or two alleles in one repetition. *Amp4* contained five AFLP phenotypes, and only one also had a different SSR genotype in one repetition.

**Table 5:** Genotypic variability indices for AFLP analyses across investigated apomictic *Taraxacum* accessions.

Accession	*amp1*	*amp2*	*amp3*	*amp4*	*amp5*	*amp6*	*O*	*S*	*P*
*N*	11	11	11	11	11	11	10	10	10
*NB*	65	64	67	64	65	64	77.5	72	78
*NPB*	31	10	21	15	20	9	3	5	13
*P (%)*	19.1	6.2	13	9.3	12.3	5.6	1.9	3.1	8
*PrB*	6	3	6	2	8	5	10	3	12
*DB*	0	2	6	1	3	4	10	3	7
*H_j_*	0.056	0.025	0.053	0.030	0.042	0.021	0.007	0.008	0.028
*NG*	10	3	10	5	3	3	1	1	3
*GD*	0.98	0.47	0.98	0.62	0.56	0.35	0.00	0.00	0.38

*N*, number of samples; *NB* – mean number of bands; *NPB* – number of polymorphic bands; *P* – polymorphism; *PrB* – number of private bands; *DB* – number of diagnostic bands; *H_j_*, gene diversity; *NG*, number of genotypes; *GD*, genotype diversity.

For taxon abbreviations see Table S1.

Polymorphism was low, ranging from the highest value of 19.1% for *amp1* to the lowest value of only 1.9% for *O*. For each apomictic accession, private and diagnostic markers were detected, only for *amp1* no diagnostic marker was observed (Table 5). Gene diversity *H_j_* was very low: *H_j_*  = 0.007– *O*; *H_j_*  = 0.008– *S* and *H_j_*  = 0.028– *P*. The highest gene diversity was observed for *amp1* (*H_j_*  = 0.056) and *amp3* (*H_j_*  = 0.053). The distribution of genotypic variability revealed that the majority of the variability, 86.6%, occurred among apomictic accessions, while only 13.4% occurred within accessions. The addition of one more hierarchical level (*AMP*, *OSP*) did not change the variability distribution, with 51.6% variability among apomictic accessions and 37.6% among groups (Table 6).

**Table 6:** Distribution of molecular variance (AMOVA) compared among molecular markers and different hierarchy of studied groups of apomictic *Taraxacum* accessions.

	Marker	Among	Among apo	Within apo	
	system	groups (%)	accessions (%)	accessions (%)	*F*_ST_*
All apomictic accessions	AFLP		86.58	13.42	0.866*
	SSR		88.44	11.56	0.884*
*AMP+OSP*	AFLP	37.57	51.62	10.81	0.827*
	SSR	22.85	66.91	10.24	0.867*
*AMP*	AFLP		78.09	21.91	0.781*
	SSR		81.02	18.98	0.810*
*OSP*	AFLP		92.45	7.55	0.925*
	SSR		97.83	2.17	0.978*

Values of *F*_ST_ correspond to the “Among apomictic accessions” differentiation; *, significant value, P<0.05.

**Figure 2:**
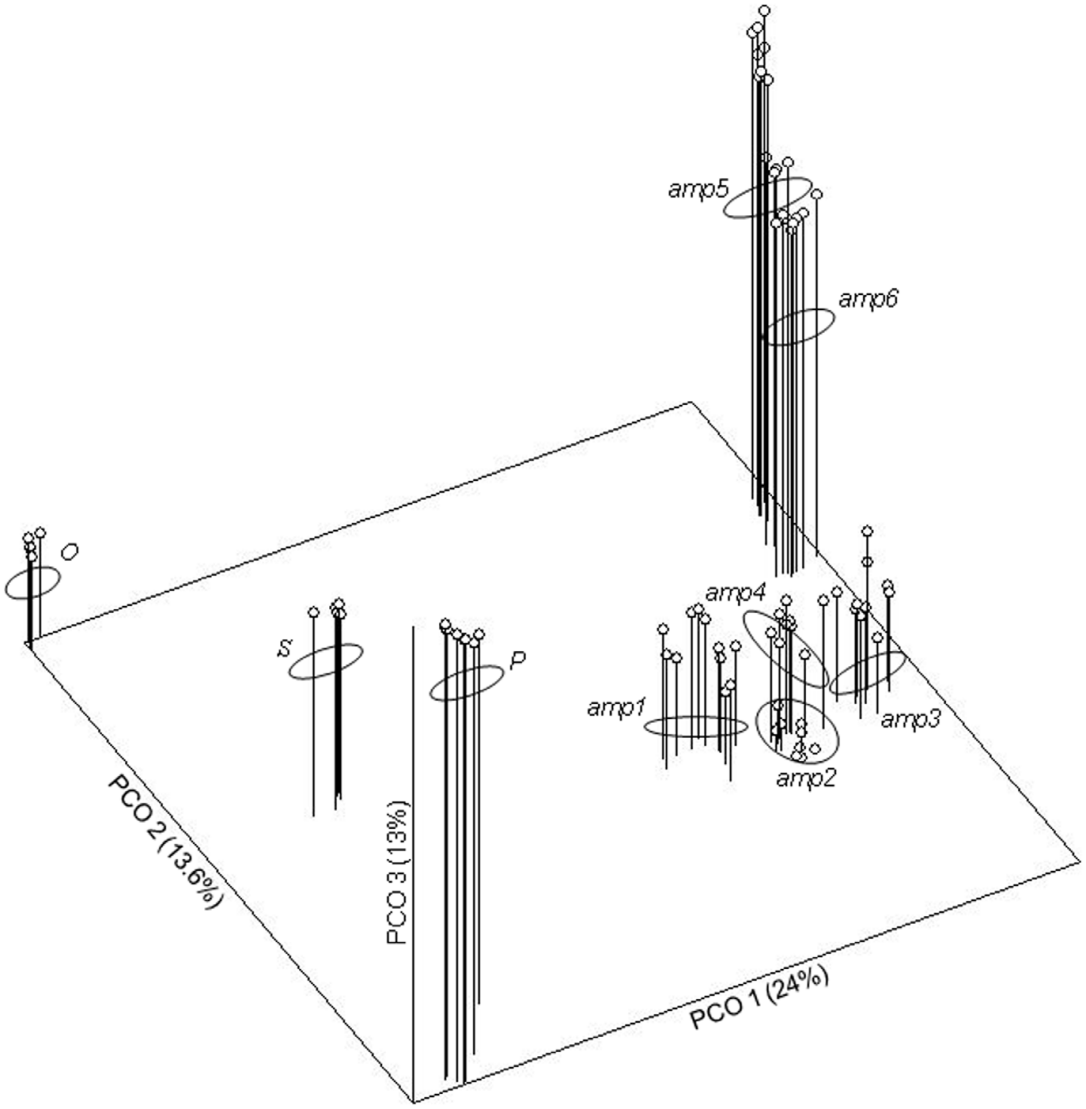
PCoA 3D plot. Principal coordinate analysis (based on Jaccard’s similarity coefficient) of 96 apomictic *Taraxacum* individuals. For taxon abbreviations see Table S1.

**Figure 3:**
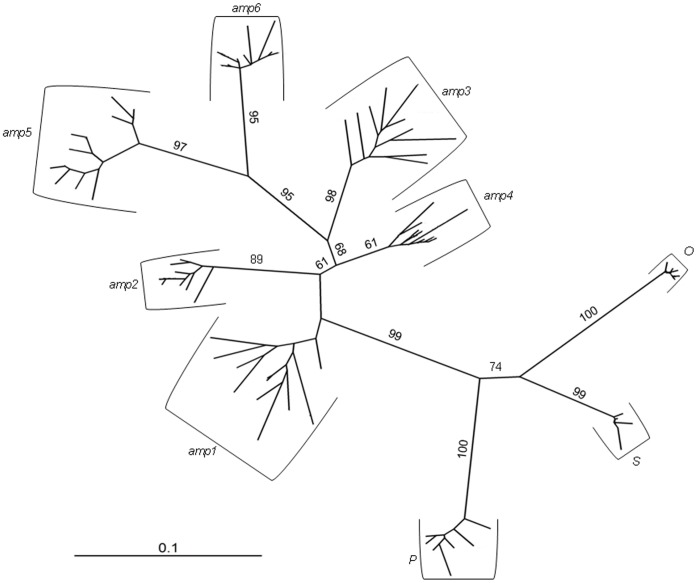
Unrooted Neighbor-joining tree. Neighbor-joining tree based on Dice coefficient of similarity (bootstrap values >50 are shown above the branches) depicting division of nine apomictic *Taraxacum* accessions into well supported agamospecific clusters (based on AFLP data of 96 individuals). For taxon abbreviations see Table S1.

**Figure 4:**
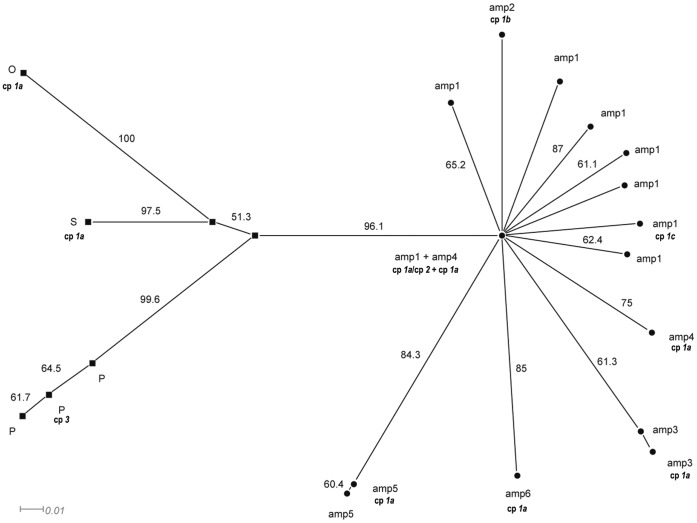
SplitDecomposition network. Network of 96 apomictic individuals of *Taraxacum* based on 162 AFLP markers (uncorrected *P*-distances; goodness of fit 85.6). Bootstrap values >50 are shown above branches. Observed cpDNA haplotypes are also designated. For taxon abbreviations see Table S1.

Principal coordinate analysis clearly discriminates all studied apomictic accessions. The first two axes of the PCoA plot discriminate between the *AMP* and *OSP* groups and place all accessions into separate groups. The third axis stressed this discrimination (Figure 2). The first three axes explain 50.6% of the variability. In the neighbour-joining tree (bootstrap support in range 61–99), apomictic accessions were grouped into nine clusters (Figure 3). All accessions are placed on highly supported branches in SplitDecomposition network (goodness of fit 85.6; Figure 4) without reticular network connections between branches. The topology of the network suggests different genetic pools between *AMP* and *OSP* and a star-like structure suggests a common origin of *AMP* group. Four AFLP-*amp1* and ten AFLP-*amp4* phenotypes were placed in the centre of this structure.

**Figure 5:**
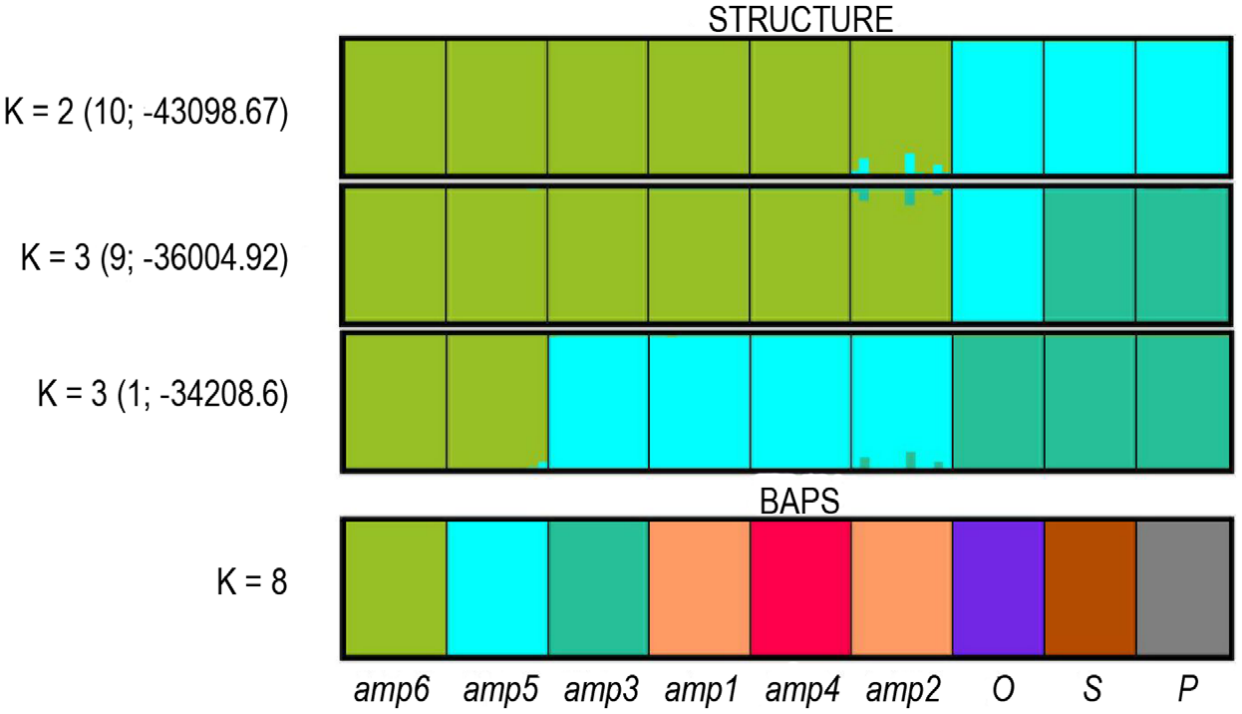
Bayesian clustering of apomictic *Taraxacum* accessions. Results of Bayesian clustering of nine apomictic *Taraxacum* accessions (96 individuals) and assignment into clusters using Structure and Baps. Each individual is represented by a vertical bar, the color representing the assignment probability to different clusters. Clustering for *K*  = 2 and *K*  = 3 (Structure ) and *K*  = 8 (Baps) are shown. The number of replicate runs producing the partition and the mean *L*(*K*) value are shown for Structure results only. Names of taxa are displayed below the graphic. For taxon abbreviations see Table S1.

The Bayesian clustering method implemented in BAPS suggested optimal partition of the samples into 8 clusters (probability equals 1) (Figure 5). The division into clusters followed microspecific insertion; only *amp1* was clustered with *amp2*. The result of Structure was unambiguous. The mean *L*(*K*) increased up to two and then flattened out. Additionally, Δ*K* showed a maximum value for *K*  = 2 (*SC*  = 1). Because a high similarity coefficient (*SC*  = 0.8) was also observed for *K*  = 3, this clustering was inspected. For the *K*  = 2, Structure identified clusters corresponded with the division of the *AMP* and *OSP* groups. For *K*  = 3, two different clustering outcomes were gained (Figure 5).

### cpDNA (trnL-trnF)

The length of the final alignment was 384 bp. Three samples were discarded from the alignment (1-*amp1*, 1-*amp6*, 1-*P*) because of high background noise in the sequences. Only five haplotypes were observed within accessions (Table 7, Table S1). Two of them were accession specific (cp*1b* for *amp2* and cp*3* for *P*), while two appeared only once and were individual specific (cp*1c* and cp*2* within *amp1*). The most common haplotype, cp*1a*, was shared among the rest of the accessions and diploids. The differences between each haplotype and the most common haplotype, cp*1a*, were as follows: substitution for cp*1b*, one deletion; for cp*1c*, nine substitutions and two deletions; and for cp*3*, the insertion of 9 bp, six substitutions, and one deletion.

**Table 7:** Comparison of cpDNA haplotypes (*trnL-trnF*) in apomictic *Taraxacum* accessions.

	0	0	0	0	1	1	1	1	1	1	1	1	3	3	3	3	3	3	3	3	3	3	3	
	6	9	9	9	0	0	0	0	0	0	0	6	3	3	4	5	5	6	6	6	7	7	8	
haplotype	4	7	8	9	0	1	2	3	4	5	6	0	7	8	9	3	4	0	2	4	0	7	1	accession presence
cp*1a*	A	*	*	*	*	*	*	*	*	*	G	G	G	T	C	T	G	G	A	A	G	C	T	*amp1, amp3, amp4, amp5, amp6, O, S, 2x*
cp*1b*	·	*	*	*	*	*	*	*	*	*	A	·	·	·	·	·	·	·	·	·	·	·	·	*amp2*
cp*1c*	*	*	*	*	*	*	*	*	*	*	·	·	·	·	·	·	·	·	·	·	·	·	·	*amp1*
cp*2*	·	*	*	*	*	*	*	*	*	*	·	T	C	C	T	*	C	·	G	G	C	T	*	*amp1*
cp*3*	·	A	T	T	A	C	A	A	A	T	·	T	C	C	·	·	·	A	·	G	·	T	*	*P*

Numbers refer to variable position observed in final alignment; * – represent insertion/deletion; · – represent match with cp*1a* haplotype;

2x – diploid sexual. For taxon abbreviations see Table S1.

## Discussion

Apomictic dandelions (with diplospory as the prevailing reproduction mode) are widely distributed in temperate zones of the Northern hemisphere. These apomicts usually predominate in populations in cooler regions and the genus as a whole is successful in colonizing great areas. In this study, we tested whether morphologically uniform phenotypes (i.e., clones, microspecies) are genetically uniform or diverged. Our data showed that apomictic accessions are genetically highly homogeneous and that its low genotypic variability can be explained by the accumulation of mutations during their asexual history. Many of the observed genotypes were clonal and accession specific and therefore accessions could be considered to represent apomictic clones. However, the variability among accessions is much higher than within accession variability. It originates from the recombination events – sexual process between apomictic pollen donor-apomictic father and sexual mother. The pattern of variability and its distribution is in correspondence with the morphological and taxonomic differentiation of accessions into apomictic microspecies.

### Source of Genotypic Variation in Apomictic Clones

Although obligate apomicts undergo only clonal reproduction, the possibility to generate genetic variability still exists. Richards [13] discussed processes of mutational changes to DNA and their accumulation (including changes to genes involved in regulation of apomixis) and gross changes at the level of chromosomes including somatic recombination or disjunctional accidents. The mutation accumulation and the recombination during female meiosis are well supported by experiments [32], [77]. Our data suggest that intraclonal diversity of *Taraxacum* apomicts is caused by mutation load within a single clone but that interclonal diversity is most likely of sexual origin. We found that all of the investigated apomictic accessions contained nearly identical genotypes or a low number of different genotypes (Table 2, Table 4, Table 5). The genotypic differentiation within accessions, in addition to *amp1*, is mutational rather than recombinational in nature (Table 4). Considering the sampling strategy and the sampling area (Figure 1), the detection of such high clonality with mutational diversity is a good representation of the asexual history of accessions [78].

Apomictic genotypes in the absence of sexual partners become frozen for hybridisation [4], [7]. Mutations therefore start to play an important role in generating the variability in clonal lineages. Such an evidence was made in asexual *Ranunculus carpaticola*, which has high allelic variation of mutational origin [11]. Similarly, populations of hexaploid apomictic *Potentilla argentea* have high variability within AFLP phenotypes indicating its mutational origin [79].

### Genotypic Diversity of Apomictic Dandelions

We found a perfect correlation between genotype fingerprints and phenotypes. All of the observed genotypes were accession specific, with no genotype shared among them. A similar pattern of genotypic diversity was observed within both markers examined in this study. With AFLPs, we detected nearly as many AFLP phenotypes as observed individuals for the *amp1* and *amp3* accessions (Table 5). Despite the high mutation rate of microsatellites (10^−2^–10^−6^; [80]), the accession always displayed fewer genotypes for SSR loci than individuals examined (Table 2, Table 4). Microsatellites detected higher clonality (low number of genotypes) within studied accessions when compared to AFLPs. A comparable pattern is known to occur in apomictic populations of aposporous *Crataegus douglasii* (*Rosaceae*) complex, where lower number of genotypes than AFLP phenotypes was detected by 13 SSR loci [12]. In contrast, *Ranunculus carpaticola* had as many genotypes as individuals for two SSR loci [11]. This contrasting pattern can be explained by the differences in mutational rates of SSR loci and the differences in marker resolution [81]. The overall genotypic diversity and polymorphism observed with AFLPs was low (Table 5). A possible explanation is the recent origin of the investigated accessions, with a minimum gained genotypic diversity, as was also proposed for *Taraxacum albidum* [31] and for the apomictic *R. carpaticola* [11]. However, the observed multilocus and locus allelic diversity assessed by SSRs was high (Table 1, Table 2) due to fixed heterozygosity in apomicts [78].

The terminology used for clones varies among geneticists, plant systematists and molecular biologists. For taxonomists or ecologists a clone usually refers to morphologically identical offspring of vegetative (or apomictic origin), whereas strictly genetic view differs in considering each unique genotype or AFLP phenotype as distinct clone even they are morphologically identical. Our data support the hierarchy of apomictic clones discussed by van Dijk *et al*. [9] who reflects different origin of clones that can be consisted of several clone mates differing in their genotypes. We similarly consider a group of individuals sharing the same AFLP phenotype and the same SSR genotype to be an asexual clone. Individuals having the same AFLP phenotype and different (but similar) SSR genotypes (or vice versa) are considered to be clone mates. They differ from each other due to different mutation loads. The lowest level in this hierarchy represents asexual lineages, i.e., individuals having unique AFLP phenotypes and SSR genotypes. This asexual lineage has its origin in clonal genotypes with variability from three possible sources: i) somatic recombination [13], ii) recombination during restitutional meiosis [77], or iii) the sexual process [25], [82]. This hierarchy takes into consideration both, the biological background of apomicts (reproduction, morphology, ecology etc.) as well as the nature of markers.

### Distribution of Genotypic Diversity

Genotypic variability in the morphological groups of apomictic dandelions that we studied had similar distributions for both types of markers. Analysis on several hierarchical levels showed that diversity is distributed mainly among accessions, whereas higher homogeneity was observed within accessions (Table 6).

Morphological groups *OSP* and *AMP* clearly form two separate pools of apomictic genotypes, which were confirmed by testing the relatedness of genotypes using several clustering methods (Figure 2, Figure 3, Figure 5). Estimated population differentiation for AFLPs/SSRs corresponds to morphological homogeneity of accessions and their genotypic variability. Apomictic accessions are highly differentiated, and are fixed for different alleles (Table 1, Table 3, Table 5). Furthermore, nearly all accessions are characterised by private markers (restricted to the group) and diagnostic markers (present in all individuals of one group) (Table 5). The only exception is *amp1*, which does not have a diagnostic marker. The high differentiation values can be expected because any mutation that will be not repaired can become fixed and frequent in clone [83]. There are no comparable results for obligate diplosporous apomicts. However, the hexaploid facultative aposporous *Potentilla argentea* and *Ranunculus carpaticola* have stronger differentiation among populations of agamospecies than within populations [11], [79]. Differences in the pattern of interpopulation differentiation can be the effect of short distance seed dispersal within these two genera. Genotypes are than concentrated on smaller geographic range and populations differ from each other. In contrast, dandelions are more effective in seed dispersal with effective spread of genotypes across wide area. In addition, reduced gene flow and differentiation through genetic drift contribute to a high diversification among populations [84].

Some microsatellite loci are highly conserved and are thus shared among individuals from distant regions. Microsatellites linked to *DIPLOSPOROUS* locus (MSTA78 and MSTA53 [17]) share the same alleles (164 bp and 202 bp, respectively) in individuals analysed from the Netherlands, Denmark and Northern Germany and thus it was hypothesised that these alleles are linked to *DIP* in natural populations of apomictic dandelions [9], [85]. Our results do not support this hypothesis, as alleles for MSTA78 and MSTA53 vary considerably in our apomictic samples. Although in some regions might be the tight linkage between microsatellite alleles and *DIP*, generally speaking it does not appear to be a general rule. The first evidence apposing this hypothesis was provided by observing a 164 bp allele (MSTA78) in population samples of sexuals from France [9].

### History of Asexual Clones

Considering the role of recombinations/mutations in pattern of genotypic variability for different apomictic accessions, in *OSP* only two genotypes do not fit the expectation for genotypes differentiating by changes gained through clonal reproduction. While the results could suggest that genotypes within the *OSP* group differ purely by accumulation of mutations, the results from cpDNA revealed that *P* had a different origin from *S* and *O* (Table 7). This incongruence is caused by overall low differentiation of SSR genotypes observed within the *OSP* group. The similarity between genotypes within the *OSP* group can also be the result of different mutational behaviour of the SSR loci within a different genetic background. Based on cpDNA results, the *OSP* group appears to be of different genetic origin than the *AMP* group. The unique haplotype cp*3* of *P* suggests that there is also a different maternal origin of this accession, while *O* and *S* share the common cp*1a* haplotype. Although *O* and *S* share the same cpDNA haplotype with the *AMP* group, both SSRs and AFLPs clearly separate all of the groups.

SplitDecomposition network does not indicate a reticulate or recombination relationship between the accessions from *AMP* group (Figure 4). All accessions are on highly supported branches, and the star-like structure of the net suggests that the *AMP* group has a unique origin (common ancestor) and a consequent radiative spreading of the clones. The possible scenario of the *AMP* group origin is the hybridisation between the sexual and apomictic pre*amp1* genotypes, in which an array of newly asexual genotypes arose and several lineages became fixed and further evolved into separate clones that share a common history. This scenario confirmed also the result of Matrix Compatibility analysis, in which nearly all genotypes are in congruence with recombinational/sexual difference of investigated accessions (Table 4). The *amp1* could represent the oldest clonal genotype within the group because it exhibits the highest variability, and it could represent the maternal haplotype for the whole group, with an SSR-allelic pattern shared within the *AMP* group and the position of *amp1* in the centre of the SplitDecomposition network. Three different cpDNA haplotypes were observed within *amp1* and four within *AMP*. The cp*1a* is the most common one; cp*1c* differs in single point deletion. Haplotype cp*2* could represent a hybridisation event with different sexual haplotypes (Table 7). The cp*1b* is specific for *amp2* and may also have a mutational origin from cp*1a*, when a single bp transition became fixed by apomixis within a clone.

The evolutionary history of *Taraxacum* shows intensive reticular evolution. Haplotypes in this study belong to group of derived haplotypes common among advanced sections [21]. Apomictic lineages can originate from multiple hybridisations of ancestral apomictic and sexual generating arrays of novel genotypes [4], [7], [19]. Apomictic genotypes become fixed in the absence of gene flow and due to genetic drift [84]. Successful clones then spread over large areas and persist for a long time [82]. A new asexual “life history” allows the clone to gain only a limited fraction of variability compared to its sexual relatives [13]. In a situation where the frequency of the sexual process is low enough, mutations become the major source of genotypic variability [86]. Clonal genotypes will produce an array of mutationally differentiated genotypes–clone mates–under such conditions [32], [36], [79]. The number of mutations will be higher in the former clones than in the younger ones. The older an asexual genotype is, the higher the rate at which mutations accumulate; this leads to the formation of a mutant genotype network.

### Conclusions

Our data demonstrate that there is both significant genetic similarity and significant differences among putative apomictic clones that were identified by phenotype. Unlike in previous studies, in which clonality was detected in a population sample of not-phenotyped accessions, we focused on the genetic structure within phenotyped apomictic accessions themselves. Both asexual life history and sexual recombination have an impact on the genetic variability of apomicts of *Taraxacum officinale* agg. From an evolutionary point of view, apomictic dandelions have undergone an asexual life history in recent evolutionary periods with preceding (sexual) hybridisation. The structure of their genotypic variability is tightly correlated with morphology. Correct genotyping is crucial requirement both in population biology and ecological studies [87]. Although detailed morphological characterisation of each single individual should be the first methodological step used in genetics, population genetics, biotechnology and biosystematic studies for reliable genotyping/sorting of clones, it is evident from our results that genotyping can significantly assist in correct determination in genera where the determination is extremely difficult.

## Supporting Information

Table S1 **List of apomictic** ***Taraxacum*** **accessions used in this study with sampling details.** Country abbreviations: CZ – Czechia; SK – Slovakia; collector abbreviations: BT – Bohumil Trávniček, RJV – Radim J. Vašut, LM – Ľuboš Majeský. The columns: F/H refers to plant material used for DNA extraction, F – fresh leaves, H – herbarium voucher, FB – flower buds; FCSS/EM refers to method used for examination of reproduction type, FCSS – Flow Cytometry Seed Screen, EM – emasculation, apo – apomictic seed formation; for *T. linearisquameum* FCM is showed where 2x  =  diploid sexual; SSR/AFLP/cpDNA shows which plants were used for SSR – microsatellite genotyping, AFLP – genotyping, cpDNA - sequencing of *trnL-trnF* region with observed haplotype + GeneBank accession number; b – double sample. Asterisk (*) indicates accessions recognized by taxonomists as validly described microspecies; absence of asterisk indicates distinct morphological groups but formally undescribed accessions (mentioned under “work names”).
(DOC)
