# Supplementary material for: The Pattern of Genetic Variability in Apomictic Clones of Taraxacum officinale Indicates the Alternation of Asexual and Sexual Histories of Apomicts
Source: PLoS One. 2012 Aug 1;7(8):e41868. doi: 10.1371/journal.pone.0041868 (PMC3411577; doi:10.1371/journal.pone.0041868)
Supplement: Table S1 — List of apomictic Taraxacum accessions used in this study with sampling details. Country abbreviations: CZ – Czechia; SK – Slovakia; collector abbreviations: BT – Bohumil Trávniček, RJV – Radim J. Vašut, LM – Ľuboš Majeský. The columns: F/H refers to plant material used for DNA extraction, F – fresh leaves, H – herbarium voucher, FB – flower buds; FCSS/EM refers to method used for examination of reproduction type, FCSS – Flow Cytometry Seed Screen, EM – emasculation, apo – apomictic seed formation; for T. linearisquameum FCM is showed where 2x = diploid sexual; SSR/AFLP/cpDNA shows which plants were used for SSR – microsatellite genotyping, AFLP – genotyping, cpDNA - sequencing of trnL-trnF region with observed haplotype + GeneBank accession number; b – double sample. Asterisk (*) indicates accessions recognized by taxonomists as validly described microspecies; absence of asterisk indicates distinct morphological groups but formally undescribed accessions (mentioned under “work names”). (DOC) [file pone.0041868.s001.doc]

**Supplemental table 1**.

List of apomictic *Taraxacum* accessions used in this study with sampling details. Country abbreviations: CZ – Czechia; SK – Slovakia; collector abbreviations: BT – Bohumil Trávniček, RJV – Radim J. Vašut, LM – Ľuboš Majeský. The columns: F/H refers to plant material used for DNA extraction, F – fresh leaves, H – herbarium voucher, FB – flower buds; FCSS/EM refers to method used for examination of reproduction type, FCSS – Flow Cytometry Seed Screen, EM – emasculation, apo – apomictic seed formation; for *T. linearisquameum* FCM is showed where 2x = diploid sexual; SSR/AFLP/cpDNA shows which plants were used for SSR – microsatellite genotyping, AFLP – genotyping, cpDNA - sequencing of *trnL-trnF* region with observed haplotype + GeneBank accession number; b – double sample. Asterisk (*) indicates accessions recognized by taxonomists as validly described microspecies; absence of asterisk indicates distinct morphological groups but formally undescribed accessions (mentioned under „work names“).

| Group/Taxon/Code | Country | Locality, GPS, Date, Collector | F/H | FCSS/EM | SSR/AFLP/cpDNA |
| --- | --- | --- | --- | --- | --- |
| **Amplum agg.** |  |  |  |  |  |
| ***amp1*** |  |  |  |  |  |
| **T. amplum* Markl. |  |  |  |  |  |
| a3 | CZ | Lýsky village near Přerov town, wet meadows on the right bank of the Strhanec brook 0.5 km E of the village; 210 m a.s.l.; 49°28'49"; 17°27'50"; 3.5.1992; BT | F | apo/- | ×/-/- |
| a5 | CZ | Přerov town, roasides of the road towards Prosenice in the Žebračka wood N of the town, 210 m a.s.l.; 49°28'11"N; 17°27'49"E; 3.5.1992; BT | F | apo/- | ×/-/- |
| a840 | SK | Slavec village near Rožňava town, small meadow at the SW village margin; 48°34'55"N; 20°27'58"E; 7.5.2006; BT | H | -/- | ×/-/- |
| a844 | SK | Hronská Breznica village near Žiar nad Hronom town, meadow at the road towards Kozelník village in the valley of Jasenica brook 2 km NNE from the Rejchard hill; 625 m a.s.l.; 48°32'46"N; 19°00'11"E; 8.5.2006; BT | H | -/- | ×/×/- |
| a859 | CZ | Polesí village near Počátky town, meadows at the road towards Běleč village 1.2 km N of the village; 690 m a.s.l.; 49°18'15"N; 15°14'42"E; 13.5.2006; BT | H | -/- | ×/-/- |
| a862 | CZ | Obrataň village near Pacov town, meadow at the road towards Nechyba settlement at the S village margin; 600 m a.s.l.; 49°25'10"N ; 14°56'40"E; 13.5.2006; BT | H | -/- | ×/×/cp*1c*/  JQ696774 |
| a865 | CZ | Všechov village near Tábor town, meadow S of the road towards Dražice village near the W village margin; 480 m a.s.l.; 49°25'56"N; 14°36'51"E; 14.5.2006; BT | H | -/- | ×/×/- |
| a911 | CZ | Hartmanice village near Veselí nad Lužnicí town, lawns in the village and meadow at the E margin of the village; ca. 475 m a.s.l.; 49°12'27"N; 14°34'00"E; 22.4.2007 | H | -/- | ×/×/cp*1a*/  JQ696775 |
| a913 | CZ | Nasavrky village near Slatiňany town, lawns and meadows at the S margin of the village; 49°50'21"N; 15°48'11"E; 25.4.2007; BT | H | -/- | ×/×/- |
| a916 | CZ | Vernýřov village near Uhlířské Janovice town, lawns and roadsides in the village; 49°50'53"N; 15°09'21"E; 25.4.2007; BT | H | -/- | ×/×/cp*2/*  JQ696807 |
| a921 | SK | Kolonica village near Snina town, meadows and lawns in SE part of the village; 48°56'57"N; 22°15'51"E; 29.4.2007; BT | F | apo/- | ×/×/- |
| a932 | CZ | Radkov village near Telč town, lawns and roadsides in the village; 49°08'42"N; 15°28'31"E; 2.5.2007; BT | F | -/- | ×/×/- |
| a953 | CZ | Křenov village near Český Krumlov town, meadow at the road 0.5 km S of the village; 540 m a.s.l.; 48°49'40"N; 14°15'12"E; 10.5.2007; BT | F | apo/- | ×/-/- |
| a976/b | CZ | Jezernice village near Lipník nad Bečvou town, lawns and roadsides in centre of the village; 49°32'39"N; 17°37'34"E; 3.5.2008; BT | F/H | apo/- | ×/×/- |
| a978 | CZ | Vepice village near Milevsko town, lawns in the village; ca. 530 a.s.l.; 49°31'34"N; 14°18'00"E; 8.5.2008; BT | F | apo/- | ×/-/- |
| a989 | CZ | Křížov village near Vlašim town, lawns and meadows in W part of the village; 49°38'30"N; 14°53'39"E; 11.5.2008; BT | F | -/- | ×/-/- |
| a995 | CZ | Ponikev village near Konice town, lawns and roadsides in the village; 49°37'29"N; 16°53'01"E; 14.5.2008; BT | F | -/- | ×/-/- |
| a996 | CZ | Studená Loučka village near Mohelnice town, lawns and roadsides in the village; 49°46'07"N; 16°49'04"E; 14.5.2008; BT | F | apo/- | ×/-/- |
| a999 | CZ | Koclířov village near Svitavy town, lawns in E part of the village (E of centre of the village); 49°45'49"N; 16°33'06"E; 14.5.2008; BT | F | apo/- | ×/-/- |
| a1000 | CZ | Pohledec village near Nové Město na Moravě town, lawns and roadsides in N part of the village; 49°34'43"N; 16°05'59"E; 14.5.2008; BT | F | apo/- | ×/×/- |
| aK07 | CZ | Kojetín town near Kroměříž town, grassy places in gardens near the eastern town margin; 49°20'54"N; 17°18'31"E; 2007; BT | F | apo/- | ×/×/- |
| aK08 | CZ | Kojetín town near Kroměříž town, grassy places in gardens near the eastern town margin; 49°20'54"N; 17°18'31"E; 2008; BT | F | -/- | ×/-/- |
| ***amp2*** |  |  |  |  |  |
| *T. albocarpaticum* |  |  |  |  |  |
| ined. |
| abc834 | SK | Pstruša village near Detva town, lawns and roadsides near car park at the main road 1.6 km E of the village; 48°32'43"N; 19°20'20"E; 6.5.2006; BT | H | -/- | ×/-/- |
| abc844 | SK | Hronská Breznica village near Žiar nad Hronom town, meadow at the road towards Kozelník village in the valley of Jasenica brook 2 km NNE from the Rejchard hill; ca.. 625 m a.s.l.; 48°32'46"N; 19°00'11"E; 8.5.2006; BT | H | -/- | ×/×/- |
| abc845 | SK | Sklené Teplice village near Banská Štiavnica town, small meadow at the turning towards Repište settlement (S of the villae); 48°31'16"N; 18°51'43"E; 8.5.2006; BT | H | -/- | ×/×/cp*1b*/  JQ696776 |
| abc847 | SK | Horná Ves village near Partizánske town, meadow at the road towards Veĺké Pole village SE of the village; 48°36'01"N; 18°30'11"E; 8.5.2006; BT | H | -/- | ×/×/ cp*1b*/  JQ696777 |
| abc852 | CZ | Hroznětín village near Ledeč nad Sázavou town, meadow N of the road towards Tunochody village (near farm); 49°45'25"N; 15°20'25"E; 10.5. 2006; BT | H | -/- | ×/×/ cp*1b*/  JQ696778 |
| abc853 | CZ | Miřátky village, meadows S of the road towards Habry town 1.3 km NW (-WNW) od the village (near Jiříkovský potok brook); 49°44'49"N; 15°30'40"E; 10.5.2006; BT | H | -/- | ×/×/- |
| abc857 | CZ | Červená Lhota village near Kardašova Řečice town, lawns at the car park in the village; ca.. 490 m a.s.l.; 49°14'56"N; 14°53'00"E; 13.5.2006; BT | H | -/- | ×/-/- |
| abc864 | CZ | Chýnov town, meadow N of the road towards Tábor town village near the SW town margin; ca.. 470 m a.s.l.; 49°24'15"N; 14°48'01"E; 14.5.2006; BT | H | -/- | ×/×/- |

| Group/Taxon/Code | Country | Locality, GPS, Date, Collector | F/H | FCSS/EM | SSR/AFLP/cpDNA |
| --- | --- | --- | --- | --- | --- |
| abc902 | CZ | Pístina village near Třeboň town, lawns in the village; ca.. 465 m a.s.l.; 49°03'01"N; 14°54'01"E; 21.4.2007; BT | H | -/- | ×/-/- |
| abc904 | CZ | Rapšach village near Třeboň town, lawns and roadsides in centre of the village; ca.. 480 M a.s.l.; 48°52'44"N; 14°55'58"E; 21.4.2007; BT | H | -/- | ×/-/- |
| abc905 | CZ | Šalmanovice village near Třeboň town, lawns and roadsides in the village; ca.. 480 m a.s.l.; 48°53'04"N; 14°46'26"E; 21.4.2007; BT | F | apo/- | ×/×/- |
| abc906 | CZ | Hrachoviště village near Třeboň town, lawns in the village; ca.. 460 M a.s.l.; 48°55'43"N; 14°46'07"E; 21.4.2007; BT | H | -/- | ×/-/- |
| abc907 | CZ | Borovany town, lawns at the railway station of Borovany, ca.. 485 m a.s.l.; 48°53'32"N; 14°38'39"E; 22.4.2007; BT | H | -/- | ×/-/- |
| abc908 | CZ | Ledenice village near České Budějovice town, lawns in E part of the village; ca.. 485 m a.s.l.; 48°56'05"N; 14°37'25"E; 22.4.2007; BT | H | -/- | ×/-/- |
| abc909 | CZ | Lišov village near České Budějovice town, lawns in S part of the village; ca.. 525 m. a.s.l.; 49°00'54"N; 14°36'43"E; 22.4.2007; BT | H | -/- | ×/×/- |
| abc910 | CZ | Neplachov village near Veselí nad Lužnicí town, meadow at the S margin of the village; ca.. 460 m a.s.l.; 49°07'35"N; 14°36'05"E;  22.4.2007; BT | H | -/- | ×/-/- |
| abc911 | CZ | Hartmanice village near Veselí nad Lužnicí town, lawns in the village and meadow at the E margin of the village; ca.. 475 m a.s.l.; 49°12'27"N; 14°34'00"E; 22.4.2007; BT | H | -/- | ×/×/- |
| abc936 | CZ | Velichov village near Ostrov town, lawns in centre of the village; 50°16'58"N; 13°00'34"E; 5.5.2007; BT | H | -/- | ×/-/- |
| abc945 | CZ | Dobrá Voda village near Nové Hrady town, lawns and roadsides in the village; ca.. 693 m a.s.l.; 48°44'27"N; 14°43'26"E; 9.5.2007; BT | H | -/- | ×/×/- |
| abc973 | CZ | Biskupice village near Luhačovice town, lawns and roadsides in centre of the village; 49°05'00"N; 17°42'35"E; 3.5.2008; BT | F | -/- | ×/-/- |
| abc974 | CZ | Slopné village near Luhačovice town, meadow at the brook at S margin of the Podvesí settlement (SSW of the village);  49°08'55"N; 17°50'43"E; 3.5.2008; BT | F | -/- | ×/×/ cp*1b*/  JQ696779 |
| abc975 | CZ | Lešná village near Valašské Meziříčí town, lawns in park and in centre of the village; 49°31'06"N; 17°55'51"E; 3.5.2008; BT | H | -/- | ×/-/- |
| cfabc846 | SK | Žarnovická Huta village near Žarnovica town, meadow N of the road towards Horné Háme village; 48°29'41"N; 18°40'52"E; 8.5.2006; BT | H | -/- | ×/-/- |
| ***amp3*** |  |  |  |  |  |
| *T. adversilobum* |  |  |  |  |  |
| ined. |
| ad821/b | CZ | Předměřice nad Jizerou village near Benátky nad Jizerou town, small meadow N of the road towards Tuřice village; 50°15'10"N; 14°°46'32"E; 1.5.2006; BT | H | -/- | ×/×/cp*1a*/  JQ696780 |
| ad824 | CZ | Mrzky village near Český Brod town, lawns and roadsides at the S village margin; 50°02'34"N; 14°48'26"E; 1.5.2006; BT | H | -/- | ×/×/- |
| ad836 | SK | Korytárky settlement near Detva town, wet meadow between road and Slatina brook; 48°32'43"N; 19°27'45"E; 6.5.2006; BT | H | -/- | ×/-/- |
| ad846/b | SK | Žarnovická Huta village near Žarnovica town, meadow N of the road towards Horné Háme village; 48°29'41"N; 18°40'52"E;  8.5.2006; BT | H | -/- | ×/×/- |
| ad860/b | CZ | Pelec village near Kamenice nad Lipou town, lawns at the small pond in the village centre; 660 m a.s.l.; 49°19'09"N; 15°08'25"E; 3.5.2006; BT | H | -/- | ×/×/- |
| ad945 | CZ | Dobrá Voda village near Nové Hrady town, lawns and roadsides in the village; 693 m a.s.l.; 48°44'27"N; 14°43'26"E; 9.5.2007; BT | H | -/- | ×/-/- |
| ad972 | CZ | Kostelec u Holešova village near Holešov town, lawns and roadsides at E margin of the village (at the road towards Roštění village); 49°22'21“N; 17°30'58“E; 3.5.2008; BT | H | -/- | ×/×/cp*1a*/  JQ696781 |
| ad995 | CZ | Ponikev village near Konice town, lawns and roadsides in the village; 49°37'29"N; 16°53'01"E; 14.5.2008; BT | F | apo/- | ×/×/cp*1a*/  JQ696782 |
| ad997 | CZ | Dlouhá Ves village near Zábřeh town, lawns and roadsides in the village; 49°49'44"N; 16°47'47"E; 14.5.2008; BT | F | -/- | ×/×/- |
| ad999 | CZ | Koclířov village near Svitavy town, lawns in E part of the village (E of centre of the village); 49°45'49"N; 16°33'06"E; 14.5.2008; BT | F | -/- | ×/×/- |
| adK07/b | CZ | Kojetín town near Kroměříž town, grassy places in gardens near the eastern town margin; 49°20'54"N; 17°18'31"E; 2007; BT | F/H | -/apo | ×/×/cp*1a*/  JQ696783 |
| adK08 | CZ | Kojetín town near Kroměříž town, grassy places in gardens near the eastern town margin; 49°20'54"N; 17°18'31"E; 2008; BT | F | -/apo | ×/×/- |
| adKZ08 | CZ | Kojetín town near Kroměříž town, grassy places in gardens near the eastern town margin; 49°20'54"N; 17°18'31"E; 2008; BT | F | apo/- | ×/×/- |
| cfad857 | CZ | Červená Lhota village near Kardašova Řečice town, lawns at the car park in the village; 490 m a.s.l.; 49°14'56"N; 14°53'00"E; 13.5.2006; BT | H | -/- | ×/-/- |
| ***amp4*** |  |  |  |  |  |
| **T. jugiferum* |  |  |  |  |  |
| H. Oellg. |
| j835 | SK | Hriňová village near Detva town, meadows at road 1 km SSE from the Javorinka hill (918 m a.s.l.) NNW of the village (in the Horná Riečka settlement); 48°36'11"N; 19°31'17"E; 6.5.2006; BT | H | -/- | ×/×/- |
| j904 | CZ | Rapšach village near Třeboň town, lawns and roadsides in centre of the village; ca. 480 m a.s.l.; 48°52'44"N; 14°55'58"E; 21.4.2007; BT | H | -/- | ×/×/cp*1a*/  JQ696784 |
| j908 | CZ | Ledenice village near České Budějovice town, lawns in E part of the village; ca. 485 m a.s.l.; 48°56'05"N; 14°37'25"E; 22.4.2007; BT | H | -/- | ×/×/- |

| Group/Taxon/Code | Country | Locality, GPS, Date, Collector | F/H | FCSS/EM | SSR/AFLP/cpDNA |
| --- | --- | --- | --- | --- | --- |
| j911 | CZ | Hartmanice village near Veselí nad Lužnicí town, lawns in the village and meadow at the E margin of the village; ca. 475 m a.s.l.; 49°12'27"N; 14°34'00"E; 22.4.2007; BT | H | -/- | ×/-/- |
| j915 | CZ | Damírov village near Golčův Jeníkov town, lawns and roadsides in the village; 49°48'42"N; 15°19'18"E; 25.4. 2007; BT | H | -/- | ×/×/cp*1a*/  JQ696785 |
| j916 | CZ | Vernýřov village near Uhlířské Janovice town, lawns and roadsides in the village; 49°50'53"N; 15°09'21"E; 25.4.2007; BT | F | apo/apo | ×/×/- |
| j972 | CZ | Kostelec u Holešova village near Holešov town, lawns and roadsides at E margin of the village (at the road towards Roštění village);  49°22'21"N; 17°30'58"E; 3.5.2008; BT | F | -/- | ×/×/- |
| j988 | CZ | Nové Práchňany near Vlašim town, lawns in the village and meadow at the W margin of the village; 49°36'10"N; 15°01'00"E; 11.5.2008; BT | F | -/- | ×/×/- |
| j989 | CZ | Křížov village near Vlašim town, lawns and meadows in W part of the village; 49°38'30"N; 14°53'39"E; 11.5.2008; BT | F | apo/- | ×/×/cp*1a*/  JQ696786 |
| j993 | CZ | Trhový Štěpánov village near Vlašim town, lawns and medaow near the railway station; 49°42'29"N; 15°00'31"E; 11.5.2008; BT | F | apo/- | ×/×/cp*1a*/  JQ696787 |
| j995 | CZ | Ponikev village near Konice town, lawns and roadsides in the village; 49°37'29"N; 16°53'01"E; 14.5.2008; BT | F | apo/- | ×/×/- |
| jKZ08 | CZ | Kojetín town near Kroměříž town, grassy places in gardens near the eastern town margin; 49°20'54"N; 17°18'31"E; 2008; BT | F | apo/- | ×/×/- |
| ***amp5*** |  |  |  |  |  |
| *T. suchovense* ined. |  |  |  |  |  |
| cfsu424 | CZ | Držková village near Fryšták town, small meadows between the road and the brook near the water reservoir at the NE margin of the village; 49°19'23"N; 17°47'41"E; 9.5.1999; BT | H | -/- | ×/-/- |
| cfsu466 | CZ | Tasov village near Veselí nad Moravou town, lawns along the brook in the W part of the village; 48°54'24"N; 17°25'34"E; 25.4.2000; BT | H | -/- | ×/-/- |
| cfsu616 | CZ | Morávka village near Frýdlant nad Ostravicí town, meadow at the road in the Morávka valley near the Mituří settlement; 49°32'48"N;  18°33'02"E; 16.5.2002; BT | H | -/- | ×/-/- |
| su469 | CZ | Nivnice village near Uherský Brod town, lawns in small park on the bank of Nivnička brook in SW part of the village; 48°58'34"N;  17°38'28"E; 25.4.2000; BT | H | -/- | ×/×/cp*1a*/  JQ696788 |
| su471 | CZ | Prusy village near Přerov town, meadow between the road Přerov - Dřevohostice and the fishpond 0.7 km NNW of the village; 49°25'57"N; 17°30'46"E; 27.4.2000; BT | H | -/- | ×/×/- |
| su474 | CZ | Špičky village near Hranice na Moravě town, meadows between the railway and the road 1.3 km W (WSW) from the Špičky railway station; 49°32'06"N; 17°47'33"E; 27.4.2000; BT | H | -/- | ×/×/- |
| su524 | CZ | Nítkovice village near Kroměříž town, lawns at the crossroad in the village; 49°12'14"N; 17°09'58"E; 1.5.2001; BT | H | -/- | ×/×/cp*1a*/  JQ696789 |
| su650 | SK | Blatnica village near Martin town, lawns in the S part of the village; 48°56'26"N; 18°55'36"E; 8.5.2003; BT | H | -/- | ×/×/- |
| su735 | SK | Maslovenka settlement N of the Turzovka town, meadow at the brook S of the road towards Hrubý Buk settlement 1 km E from the church in the Hrubý Buk settlement; 49°28'01"N; 18°38'29"E; 14.5.2004; BT & RJV | H | -/- | ×/×/- |
| su776 | SK | Korňa village near Turzovka town, lawns near brook 0.5 km E of the church in the village; 49°24'50"N; 18°33'07"E; 10.5.2005; BT | H | -/- | ×/×/- |
| su783 |  | Dolní Lhota village, W of Ostrava town, small meadow at the brook near crossroad in the S part of the village; 49°50'12"N; 18°05'16"E;  11.5.2005;BT | H | -/- | ×/×/cp*1a*/  JQ696790 |
| su846 | SK | Žarnovická Huta village near Žarnovica town, meadow N of the road towards Horné Háme village; 48°29'41"N; 18°40'52"E; 8.5.2006; BT | H | -/- | ×/×/cp*1a*/  JQ696791 |
| su847 | SK | Horná Ves village near Partizánske town, meadow at the road towards Veľké Pole village SE of the village; 48°36'01"N; 18°30'11"E;  8.5.2006; BT | H | -/- | ×/×/- |
| su973 | CZ | Biskupice village near Luhačovice town, lawns and roadsides in centre of the village; 49°05'00"N; 17°42'35"E; 3.5.2008; BT | F | apo/- | ×/×/- |
| ***amp6*** |  |  |  |  |  |
| *T. jari-cimrmanii* |  |  |  |  |  |
| ined. |
| tp813 | CZ | Dlouhopolsko village near Městec Králové town, meadow between the road towards Žehuň village and Dlouhopolský rybník fishpond near the SW village margin; 50°10'24"N; 15°18'08"E; 28.4.2006; BT | H | -/- | ×/-/- |
| tp832 | CZ | Jarpice village near Slaný town, meadow N of the road towards Horní Kamenice village at the W village margin; 50°19'13"N; 14°04'50"E; 3.5.2006; BT | H | -/- | ×/×/- |

| Group/Taxon/Code | Country | Locality, GPS, Date, Collector | F/H | FCSS/EM | SSR/AFLP/cpDNA |
| --- | --- | --- | --- | --- | --- |
| tp835 | SK | Hriňová village near Detva town, meadows at road 1 km SSE from the Javorinka hill (918 m a.s.l.) NNW of the village (in the Horná Riečka settlement); 48°36'11"N; 19°31'17"E; 6.5.2006; BT | H | -/- | ×/-/- |
| tp836 | SK | Korytárky settlement near Detva town, wet meadow between road and Slatina brook; 48°32'43"N; 19°27'45"E; 6.5.2006; BT | H | -/- | ×/-/- |
| tp842 | SK | Muráň village near Revúca town, meadow near SW village margin; 48°44'20"N; 20°02'21"E; 7.5.2006; BT | H | -/- | ×/×/- |
| tp846 | SK | Žarnovická Huta village near Žarnovica town, meadow N of the road towards Horné Hámre village; 48°29'41"N; 18°40'52"E;  8.5.2006; BT | H | -/- | ×/-/- |
| tp847 | SK | Horná Ves village near Partizánske town, meadow at the road towards Veľké Pole village SE of the village; 48°36'01"N; 18°30'11"E; 8.5.2006; BT | H | -/- | ×/-/- |
| tp849 | CZ | Babice village near Světlá nad Sázavou town, meadows along Sázava river at the E village margin; 49°37'59"N; 15°28'38"E; 10.5.2006; BT | H | -/- | ×/-/- |
| tp851 | CZ | Hostkovice village near Ledeč nad Sázavou town, lawns in the village; 49°46'12"N; 15°17'29"E; 10.5.2006; BT; | H | -/- | ×/×/- |
| tp860 | CZ | Pelec village near Kamenice nad Lipou town, lawns at the small pond in the village centre, 660 m a.s.l.; 49°19'09"N; 15°08'25"E; 13.5.2006; BT | H | -/- | ×/×/- |
| tp864 | CZ | Chýnov town, meadow N of the road towards Tábor town village near the SW town margin, 470 m a.s.l.; 49°24'15"N; 14°48'01"E; 14.5.2006; BT | H | -/- | ×/-/- |
| tp865 | CZ | Všechov village near Tábor town, meadow S of the road towards Dražice village near the W village margin; 480 m a.s.l.; 49°25'56"N; 14°36'51"E; 14. 5.2006; BT | H | -/- | ×/×/- |
| tp869 | CZ | Chotěvice village near Hostinné town, lawns and small meadows at the road towards Pilníkov village 0.7 km ENE from the turning towards Čermná village; 50°31'30"N; 15°47'12"E; 17.5.2006; BT | H | -/- | ×/-/- |
| tp891 | CZ | Židněves village near Mladá Boleslav town, meadow at the SE margin of the village; 50°24'42"N; 15°00'05"E; 15.4.2007; BT | H | -/- | ×/×/cp*1a*/  JQ696792 |
| tp893 | CZ | Strašov village near Přelouč town, meadows at the Strašovský rybník pond 1.1 km N of the village; 50°05'48"N; 15°31'26"E; 18.4.2007; BT | H | -/- | ×/-/- |
| tp897 | CZ | Černilov village near Hradec Králové town, lawns and roadsides in NW part of the village; 50°16'11"N; 15°54'22"E; 18.4.2007; BT | H | -/- | ×/-/- |
| tp903 | CZ | Lutová village near Třeboň town, lawns in the village, ca 460 m a.s.l.; 48°59'23"N; 14°54'32"E; 21.4.2007; BT | H | -/- | ×/×/- |
| tp906 | CZ | Hrachoviště village near Třeboň town, lawns in the village, ca 460 M a.s.l.; 48°55'43"N; 14°46'07"E; 21.4.2007; BT | H | -/- | ×/-/- |
| tp910 | CZ | Neplachov village near Veselí nad Lužnicí town, meadow at the S margin of the village, ca 460 m a.s.l.; 49°07'35"N; 14°36'05"E; 22.4.2007; BT | H | -/- | ×/×/- |
| tp966 | CZ | Lužice village near Most town, lawns in N part of the village, ca 260 m a.s.l.; 50°29'36"N; 13°45'09"E; 19.4.2008; BT | H | -/- | ×/-/- |
| tp972 | CZ | Kostelec u Holešova village near Holešov town, lawns and roadsides at E margin of the village (at the road towards Roštění village);  49°22'21"N; 17°30'58"E; 3.5.2008; BT | H | -/- | ×/-/- |
| tp973 | CZ | Biskupice village near Luhačovice town, lawns and roadsides in centre of the village; 49°05'00"N; 17°42'35"E; 3.5.2008; BT | F | apo/- | ×/-/- |
| tp974 | CZ | Slopné village near Luhačovice town, meadow at the brook at S margin of the Podvesí settlement (SSW of the village); 49°08'55"N; 17°50'43"E; 3.5.2008; BT | F | apo/apo | ×/-/- |
| tp975 | CZ | Lešná village near Valašské Meziříčí town, lawns in park and in centre of the village; 49°31'06"N; 17°55'51"E; 3.5. 2008; BT | F | -/- | ×/-/- |
| tp976 | CZ | Jezernice village near Lipník nad Bečvou town, lawns and roadsides in centre of the village; 49°32'39"N; 17°37'34"E; 3.5.2008; BT | H | -/- | ×/×/- |
| tp983 | CZ | Dolní Novosedly village near Písek town, lawns and roadsides in the village, ca 485 M a.s.l.; 49°19'44"N; 14°11'48"E; 9.5.2008; BT | F | apo/- | ×/-/- |
| tp985 | CZ | Koloděje nad Lužnicí village near Týn nad Vltavou town, lawns along the road in S part of the village (towards Týn nad Vltavou),  ca.. 390 m a.s.l.; 49°14'55"N; 14°25'16"E; 9.5.2008; BT | F | -/- | ×/×/cp*1a*/  JQ696793 |
| tp988 | CZ | Nové Práchňany near Vlašim town, lawns in the village and meadow at the W margin of the village; 49°36'10"N; 15°01'00"E;  11.5.2008; BT | F | -/- | ×/×/cp*1a*/  JQ696794 |
| tp989 | CZ | Křížov village near Vlašim town, lawns and meadows in W part of the village; 49°38'30"N; 14°53'39"E; 11.5.2008; BT | F | apo/- | ×/-/- |
| tp995 | CZ | Ponikev village near Konice town, lawns and roadsides in the village; 49°37'29"N; 16°53'01"E; 14.5.2008; BT | F | -/- | ×/-/- |
| tp995 | CZ | Ponikev village near Konice town, lawns and roadsides in the village; 49°37'29"N; 16°53'01"E; 14.5.2008; BT | F | -/- | ×/-/- |
| tp996/b | CZ | Studená Loučka village near Mohelnice town, lawns and roadsides in the village; 49°46'07"N; 16°49'04"E; 14.5.2008; BT | F | apo/- | ×/-/- |
| tp997 | CZ | Dlouhá Ves village near Zábřeh town, lawns and roadsides in the village; 49°49'44"N; 16°47'47"E; 14.5.2008 | F | -/- | ×/-/- |
| tp999 | CZ | Koclířov village near Svitavy town, lawns in E part of the village (E of centre of the village); 49°45'49"N; 16°33'06"E; 14.5.2008 | F | apo/- | ×/-/- |
| tpK07 | CZ | Kojetín town near Kroměříž town, grassy places in gardens near the eastern town margin; 49°20'54"N; 17°18'31"E; 2007; BT | F | -/- | ×/-/- |
| tpK08 | CZ | Kojetín town near Kroměříž town, grassy places in gardens near the eastern town margin; 49°20'54"N; 17°18'31"E; 2008; BT | F | -/- | ×/-/- |
| **OSP Group** |  |  |  |  |  |
| ***O*** |  |  |  |  |  |
| **T. obtusifrons* |  |  |  |  |  |
| Markl. |
| o1 | CZ | Lýsky village near Přerov town, small meadow near the railway underpass at the W margin of the village, 210 m a.s.l.; 49°28'52"N; 17°27'15"E; 3.5.1992; BT | H | -/- | ×/-/- |
| Group/Taxon/Code | Country | Locality, GPS, Date, Collector | F/H | FCSS/EM | SSR/AFLP/cpDNA |
| o554 | CZ | Vilémovice village near Blansko town, meadows in the valley near the crossroad 0.2 km N from the N margin of the village; 49°22'13"N; 16°44'35"E; 15.5.2001; BT | H | -/- | ×/×/cp*1a*/  JQ696799 |
| o555 | CZ | Ludíkov village near Boskovice town, small meadow at the crossroad N of the village; 49°27'30"N; 16°44'08"E; 15.5.2001;BT | H | -/- | ×/-/- |
| o558 | CZ | Úsobrno village near Jevíčko town, lawns in the S part of the village; 49°34'59"N; 16°46'00"E; 15.5.2001; BT | H | -/- | ×/×/- |
| o563 | CZ | Nový Rychnov village near Pelhřimov town, meadow near the SE margin of the village; 49°22'45"N; 15°22'18"E; 17.5.2001; BT | H | -/- | ×/-/- |
| o565 | CZ | Bílý Kámen village near Jihlava town, meadow at the road near the NE margin of the village; 49°26'13"N; 15°30'38"E; 17.5.2001; BT | H | -/- | ×/×/- |
| o576 | CZ | Plučisko settlement near Chropyně towní, small meadow at the wood near the crossroad not far from the settlement; 49°24'40"N; 17°22'17"E; 25.4.2002; BT | H | -/- | ×/×/cp*1a*/  JQ696800 |
| o632 | CZ | Prušánky village near Hodonín town, lawns near swimming poll in the NE part of the village; 48°50'02"N; 16°58'46"E; 27.4.2003; BT | H | -/- | ×/×/- |
| o632 | CZ | Prušánky village near Hodonín town, lawns near swimming poll in the NE part of the village; 48°50'02"N; 16°58'46"E; 27.4.2003; BT | H | -/- | ×/×/- |
| o650 | SK | Blatnica village near Martin town, lawns in the S part of the village; 48°56'26"N; 18°55'36"E; 8.5.2003; BT | H | -/- | ×/×/cp*1a*/  JQ696801 |
| o681 | CZ | Tovačov town, lawns and meadows in the SE part of the town; 49°25'39"N; 17°17'31"E; 26.4.2004; BT | H | -/- | ×/-/- |
| o914 | CZ | Horky village near Čáslav town, lawns in the village; 49°52'14"N; 15°26'21"E; 25.4.2007; BT | F | apo/apo | ×/-/- |
| o915/b | CZ | Damírov village near Golčův Jeníkov town, lawns and roadsides in the village; 49°48'42"N; 15°19'18"E; 25.4.2007; BT | F | -/apo | ×/×/cp*1a*/  JQ696802 |
| o932/b | CZ | Radkov village near Telč town, lawns and roadsides in the village; 49°08'42"N; 15°28'31"E; 2.5.2007; BT | F | apo/- | ×/-/- |
| o978 | CZ | Vepice village near Milevsko town, lawns in the village; ca.. 530 m.a.s.l.; 49°31'34"N; 14°18'00"E; 8.5.2008; BT | F | apo/- | ×/-/- |
| o996 | CZ | Studená Loučka village near Mohelnice town, lawns and roadsides in the village; 49°46'07"N; 16°49'04"E; 14.5.2008; BT | F | apo/- | ×/-/- |
| oH | CZ | Hutisko-Solanec village, cultivated meadow in the village centre, ca. 490 m.a.s.l.; 49°25'51.71"N; 18°13'5.19"E; 3.5.2006; RJV | F | apo/- | ×/×/- |
| oKZ08 | CZ | Kojetín town near Kroměříž town, grassy places in gardens near the eastern town margin; 49°20'54"N; 17°18'31"E; 2008; BT | F | -/- | ×/×/- |
| oVS | CZ | Vašútky settlemet, wet meadow in the settlement, ca. 680 m.a.s.l.; 49°25'0.89"N, 18°21'52.69"E; 3.5.2006; RJV | F | apo/- | ×/×/- |
| oX | CZ | Černotín village, wet meadow behind the village in direction to the Milotice nad Bečvou village, ca. 250 m.a.s.l.; 49°32'20.16"N; 17°48'3.28"E; 5.5.2009; LM | F | -/- | ×/-/- |
| ***S*** |  |  |  |  |  |
| *T. stridulum* ined. |  |  |  |  |  |
| s818 | CZ | Doubravany village near Nymburk town, lawns in the village centre; 50°18'25"N; 15°07'47"E; 1.5.2006; BT | H | -/- | ×/×/cp*1a*/  JQ696806 |
| s812 | CZ | Chlumec nad Cidlinou town, lawns and roadsides at the road towards Kladruby village near the bridge across Cidlina river; 50°08'54"N; 15°27'52"E; 28.4.2006; BT | H | -/- | ×/-/- |
| s815 | CZ | Bobnice village near Nymburk town, lawns and roadsides in the village centre; 50°13'06"N; 15°03'17"E; 28.4.2006; BT | H | -/- | ×/-/- |
| s816 | CZ | Dymokury village near Nymburk town, pasture at the road towards Záhornice village near the E village margin; 50°14'48"N; 15°12'32"E; 28.4.2006; BT | H | -/- | ×/-/- |
| s817 | CZ | Hlušice village near Nový Bydžov town, lawns and roadsides in the village; 50°15'45"N; 15°24'05"E; 28.4.2006; BT | H | -/- | ×/-/- |
| s837 | SK | Lovinobaňa village, small meadow at the road towards Lučenec town near the crossroad at the SE village margin; 48°25'49"N; 19°35'43"E; 6.5.2006; BT | H | -/- | ×/-/- |
| s868 | CZ | Radeč village near Úpice town, meadow at the road towards Starý Rokytník village near cemetery 0.5 km N of the village; 50°30'50"N; 15°59'00"E; 17.5.2006; BT | H | -/- | ×/-/- |
| s885 | SK | Gerlachov village near Poprad town, lawns and roadsides in NW part of the village; 49°05'50"N; 20°12'27"E; 26.5.2006; BT | H | -/- | ×/×/- |
| s887 | CZ | Pučery village near Zásmuky town, lawns in the village; 49°57'54"N; 15°06'22"E; 15.4.2007; BT | H | -/- | ×/×/cp*1a*/  JQ696804 |
| s891 | CZ | Židněves village near Mladá Boleslav town, meadow at the SE margin of the village; 50°24'42"N; 15°00'05"E; 15.4.2007; BT | H | -/- | ×/×/- |
| s894 | CZ | Žehušice village near Kutná Hora town, lawns and roadsides in SE part of the village; 49°58'02"N; 15°24'38"E; 18.4.2007; BT | F | -/- | ×/-/- |
| s895 | CZ | Chotělice village near Nový Bydžov town, meadow and roadsides at E margin of the village; 50°18'19"N; 15°28'04"E; 18.4.2007; BT | H | -/- | ×/×/- |
| s896 | CZ | Jeřice village near Hořice town, meadow at NW margin of the village; 50°20'44"N; 15°40'28"E; 18.4.2007; BT | H | -/- | ×/-/- |
| s897 | CZ | Černilov village near Hradec Králové town, lawns and roadsides in NW part of the village; 50°16'11"N; 15°54'22"E; 18.4.2007; BT | H | -/- | ×/×/- |
| s905 | CZ | Šalmanovice village near Třeboň town, lawns and roadsides in the village; ca.. 480 m a.s.l.; 48°53'04"N; 14°46'26"E; 21.4.2007; BT | H | -/- | ×/-/- |
| s910 | CZ | Neplachov village near Veselí nad Lužnicí town, meadow at the S margin of the village; ca.. 460 m a.s.l.; 49°07'35"N; 14°36'05"E;  22.4.2007; BT | H | -/- | ×/-/- |
| Group/Taxon/Code | Country | Locality, GPS, Date, Collector | F/H | FCSS/EM | SSR/AFLP/cpDNA |
| s911 | CZ | Hartmanice village near Veselí nad Lužnicí town, lawns in the village and meadow at the E margin of the village; ca.. 475 m a.s.l.;  49°12'27"N; 14°34'00"E; 22.4.2007; BT | H | -/- | ×/×/- |
| s912 | CZ | Osík village near Litomyšl town, lawns and roadsides in NE part of the village; 49°51'18"N; 16°17'37"E; 25.4.2007; BT | F | apo/- | ×/-/- |
| s915 | CZ | Damírov village near Golčův Jeníkov town, lawns and roadsides in the village; 49°48'42"N; 15°19'18"E; 25.4.2007; BT | H | -/- | ×/×/cp*1a*/  JQ696805 |
| s933 | CZ | Bílkov village near Dačice town, lawns and roadsides in the village; 49°05'16"N; 15°28'31"E; 2.5.2007; BT | F | apo/- | ×/×/cp*1a*/  JQ696803 |
| s982 | CZ | Zlivice village near Písek town, roadsides and lawns at the pond in the village, ca.. 425 m a.s.l.; 49°21'26"N; 14°06'16"E; 9.5.2008; BT | F | apo/- | ×/-/- |
| s983 | CZ | Dolní Novosedly village near Písek town, lawns and roadsides in the village; ca.. 485 m a.s.l.; 49°19'44"N; 14°11'48"E; 9.5.2008; BT | F | apo/apo | ×/×/- |
| s995 | CZ | Ponikev village near Konice town, lawns and roadsides in the village; 49°37'29"N; 16°53'01"E; 14.5.2008; BT | F | apo/- | ×/-/- |
| ***P*** |  |  |  |  |  |
| **T. pulchrifolium* |  |  |  |  |  |
| Markl. |
| pul555 | CZ | Ludíkov village near Boskovice town, small meadow at the crossroad N of the village; 49°27'30"N; 16°44'08"E; 15.5.2001; BT | H | -/- | ×/×/- |
| pul559 | CZ | Brněnec village near Svitavy town, lawns at the road in the W part of the village, 1.5 km W from the Březová nad Svitavou railway station; 49°38'02"N; 16°29'55"E; 15.5.2001; BT | H | -/- | ×/×/cp*3*/  JQ696808 |
| pul560 | CZ | Malé Hradisko village near Prostějov town, meadow at the road bend near the SE margin of the village; 49°29'30"N; 16°52'55"E;  17.5.2001; BT | H | -/- | ×/-/- |
| pul570 | CZ | Svratka village near Hlinsko town, meadow near the wood at the road towards Křižánky 1.4 km ESE from the main crossroad in the village; 49°42'19"N; 16°03'13"E; 20.5.2001; BT | H | -/- | ×/×/- |
| pul573 | CZ | Horní Lomná village near Jablunkov town, lawns and small meadows at the road near the Přelač settlement; 49°30'43"N; 18°39'01"E;  23.5.2001; BT | H | -/- | ×/×/- |
| pul651 | SK | Oščadnica village near Čadca town, meadow above the road 1.5 km SSE of the Liesková hill (850 m) NNE of the village; 49°28'48"N;  18°55'39"E; 8.5.2003; BT | H | -/- | ×/×/cp*3*/  JQ696809 |
| pul681 | CZ | Tovačov town, lawns and meadows in the SE part of the town; 49°25'39"N; 17°17'31"E; 26.4.2004; BT | H | -/- | ×/×/- |
| pul739 | CZ | Vrchlabí town, wet meadow S from the road towards Valteřice village N from the pond; 50°37'08"N; 15°36'14"E; 17.5.2004; BT | H | -/- | ×/×/- |
| pul750 | CZ | Bolelouc village near Tovačov town, lawns and roadsides at the E margin of the village; 49°29'32"N; 17°16'31"E; 27.4.2005; BT | H | -/- | ×/×/- |
| pul912 | CZ | Osík village near Litomyšl town, lawns and roadsides in NE part of the village; 49°51'18"N; 16°17'37"E; 25.4.2007; BT | H | -/- | ×/-/- |
| pul943/b | CZ | Zadní Chodov village near Planá town, lawns and roadsides in W part of the village (near church); 49°53'28"N; 12°39'12"E; 6.5.2007; BT | F | apo/- | ×/×/cp*3*/  JQ696810 |
| pul996 | CZ | Studená Loučka village near Mohelnice town, lawns and roadsides in the village; 49°46'07"N; 16°49'04"E; 14.5.2008; BT | F | -/- | ×/-/- |
| pulH | CZ | Hutisko-Solanec village, cultivated meadow in the village centre, ca. 490 m.a.s.l.; 49°25'51.71"N; 18°13'5.19"E; 3.5.2006; RJV | F | -/- | ×/-/- |
| pulKZ08 | CZ | Kojetín town near Kroměříž town, grassy places in gardens near the eastern town margin; 49°20'54"N; 17°18'31"E; 2008; BT | F | -/- | ×/-/- |
| pulTRE | SK | Trebichava village near Bánovce nad Bebravou town, wet meadow behind the village in the SE direction; 48°49'33"N; 18°18'24"E; 28.4.2009; LM & RJV | F | -/- | ×/×/- |
| pulX | CZ | Černotín village, wet meadow behind the village in direction to the Milotice nad Bečvou village, ca. 250 m a.s.l.; 49°32'15.97"N; 17°47'49.29"E; 5.5.2009; LM | F | -/- | ×/- |
| **T.* |  |  |  | FCM |  |
| *linearisquameum* |
| Soest |
| R1 | CZ | Klentnice village, ruderal grasses and wood pathway verges in vicinity of the castle ruins Sirotčí hrádek; ca. 400 m a. s. l.; 48°50'38.12"N; 16°38'9.75"E; 25.4.2004; RJV | FB | 2x | ×/×/ cp*1a*/  JQ696795 |
| R3 | CZ | Klentnice village, ruderal grasses and wood pathway verges in vicinity of the castle ruins Sirotčí hrádek; ca. 400 m a. s. l.; 48°50'38.12"N; 16°38'9.75"E; 25.4.2004; RJV | FB | 2x | ×/×/ cp*1a*/  JQ696796 |
| R5 | CZ | Klentnice village, ruderal grasses and wood pathway verges in vicinity of the castle ruins Sirotčí hrádek; ca. 400 m a. s. l.; 48°50'38.12"N; 16°38'9.75"E; 25.4.2004; RJV | FB | 2x | ×/×/ cp*1a*/  JQ696797 |
| R6 | CZ | Klentnice village, ruderal grasses and wood pathway verges in vicinity of the castle ruins Sirotčí hrádek; ca. 400 m a. s. l.; 48°50'38.12"N; 16°38'9.75"E; 25.4.2004; RJV | FB | 2x | ×/×/ cp*1a*/  JQ696798 |
